# Initial Characterization of the Pig Skin Bacteriome and Its Effect on *In Vitro* Models of Wound Healing

**DOI:** 10.1371/journal.pone.0166176

**Published:** 2016-11-08

**Authors:** Matthew K. McIntyre, Trent J. Peacock, Kevin S. Akers, David M. Burmeister

**Affiliations:** 1 Damage Control Resuscitation, United States Army Institute of Surgical Research, Fort Sam Houston, Texas, United States of America; 2 Dental Trauma Research Detachment, United States Army Institute of Surgical Research, Fort Sam Houston, Texas, United States of America; 3 Extremity Trauma and Regenerative Medicine, United States Army Institute of Surgical Research, Fort Sam Houston, Texas, United States of America; Universitatsklinikum Hamburg-Eppendorf, GERMANY

## Abstract

Elucidating the roles and composition of the human skin microbiome has revealed a delicate interplay between resident microbes and wound healing. Evolutionarily speaking, normal cutaneous flora likely has been selected for because it potentiates or, at minimum, does not impede wound healing. While pigs are the gold standard model for wound healing studies, the porcine skin microbiome has not been studied in detail. Herein, we performed 16S rDNA sequencing to characterize the pig skin bacteriome at several anatomical locations. Additionally, we used bacterial conditioned-media with *in vitro* techniques to examine the paracrine effects of bacterial-derived proteins on human keratinocytes (NHEK) and fibroblasts (NHDF). We found that at the phyla level, the pig skin bacteriome is similar to that of humans and largely consists of *Firmicutes* (55.6%), *Bacteroidetes* (20.8%), *Actinobacteria* (13.3%), and *Proteobacteria* (5.1%) however species-level differences between anatomical locations exist. Studies of bacterial supernatant revealed location-dependent effects on NHDF migration and NHEK apoptosis and growth factor release. These results expand the limited knowledge of the cutaneous bacteriome of healthy swine, and suggest that naturally occurring bacterial flora affects wound healing differentially depending on anatomical location. Ultimately, the pig might be considered the best surrogate for not only wound healing studies but also the cutaneous microbiome. This would not only facilitate investigations into the microbiome’s role in recovery from injury, but also provide microbial targets for enhancing or accelerating wound healing.

## Introduction

The symbiotic relationship between the cutaneous microbiome and the skin itself includes multiple prokaryotic niches (e.g., epidermis, sweat gland, sebaceous gland, hair follicle etc.) that provide protective immunity against pathogenic infections [1, 2]. The bacteriome of human skin also displays significant spatial and temporal diversity [3, 4]. A disruption of this relationship (i.e., dysbiosis), can be coupled with a loss of bacterial diversity which is associated with a number of conditions including psoriasis, atopic dermatitis, rosacea, and acne [5, 6]. Although this is also the case with diabetic wounds [7], the influence of the normal skin microbiome on cutaneous wound healing remains largely unstudied.

When injured, the skin’s main barrier function becomes compromised and the inflammatory phase of wound healing can both enhance or suppress recovery [8]. The cutaneous microbiome has been shown to modulate T-cell phenotype as well as inflammatory mediators such as IL-1, IL-10, and interferon-γ during wound healing [1, 9–12]. Moreover, when bacteria normally found in the human stratum corneum colonize a wound, antimicrobial and inflammatory proteins are released from basal keratinocytes [13]. Animal studies have recently revealed that vancomycin-treated mice exhibit dysbiosis of the cutaneous microbiome which is associated with delayed wound healing [14]. Alternatively, germ-free mice exhibit accelerated wound closure and less scar formation, which is reversed by inoculation with conventional microbiota [12]. While these conflicting studies highlight the potential impact of the microbiome on wound healing, the applicability of rodent models to humans remains controversial [15].

Porcine models are widely considered to be the gold standard for cutaneous wound healing studies due to structural and healing similarities to human skin [16–20]. As such, a large number of studies utilize pigs to examine wound healing to show, for example, that significant anatomic variability in the rates of wound healing exist [21], which is also affected by age [22]. Despite this, the pig skin microbiome is largely uncharacterized, with the only information involving changes in the porcine inner ear (i.e., pinnae) microbiome due to scabies [23]. To this end, we utilized 16S rDNA sequencing to characterize the healthy porcine skin bacteriome in terms of microbial composition, phylogeny, density, and diversity at four anatomical locations. Additionally, we examined paracrine effects of the bacterial supernatant from each of these anatomical locations on human fibroblast and keratinocyte migration, viability, and keratinocyte Vascular Endothelial Growth Factor (VEGF) release. These assays were chosen to reflect potential effects of resident bacteria on cellular motility, health, and growth factor release that are vital for normal wound healing. We hypothesized that significant anatomical variability exists in the cutaneous microbiome of the pig, which would be associated with differences in *in vitro* wound healing assays. We found significant similarities between porcine and human cutaneous bacteriomes, and found that conditioned media from different anatomical locations had drastically different effects on fibroblast and keratinocyte phenotypes.

## Materials and Methods

### Ethics

Seven-three month old female Yorkshire (*Sus Scrofa*) pigs (Midwest Research Swine) weighing between 30.2 and 39.2 kg were allowed *ad libitum* access to food and water. After a minimum of 72 hours of acclimation into the facilities, animals were swabbed for bacterial analysis during normal blood screening procedures. All animal experiments were approved by veterinary consultation within the Animal Care and Use Committee, US Army Institute of Surgical Research. This study has been conducted in compliance with the Animal Welfare Act, the implementing Animal Welfare *Regulations*, and the principles of the *Guide for the Care and Use of Laboratory Animals*.

### Swabbing

**Fig 1:**
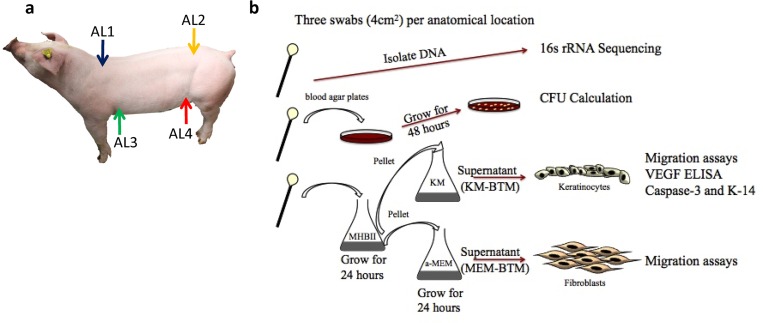
Methods Summary (a) Schematic of swabbing locations. (b) Graphical representation of methods used.

Pigs were anesthetized using telazol (IM, 4mg/kg) and swabbing was performed using cotton tipped applicators (ThermoFisher Scientific) soaked in sterile saline solution (Baxter). Four anatomical locations on each animal termed Cranial-Dorsal (AL1), Caudal-Dorsal (AL2), Cranial-Ventral (AL3), and Caudal-Ventral (AL4) were swabbed on unshaven pigs as shown in Fig 1. Dorsal and ventral swabs were taken from a location 4 cm from the vertebrae or nipple line, respectively, using a modified Levine technique covering a 4 cm^2^ area. Cranial swabs were taken just distal to the scapula, while caudal swabs were taken just proximal to the ischium in order to reflect known differences in cranial versus caudal wound healing. Each site was separated into three adjacent swabs for placement in: 1mL enzymatic lysis buffer (20mM Tris pH 8, 2mM EDTA, and 1.2% Triton X-100) stored at -80°C until 16s rDNA sequencing; 1mL saline for immediate aerobic culture for CFU estimation; and 1mL Mueller-Hinton Broth II (MHBII, BD) for bacterial expansion. Swabbing was randomized by alternating between left and right sides from animal to animal, however the side swabbed remained constant for each animal.

### DNA Isolation and Sequencing

After thawing, swabs were vortexed for 30 seconds, and 500uL of suspension was removed for extraction. DNA was bead extracted and DNA isolation was performed using the BioFire Platinum Path IT1-2-13 DNA/RNA Extraction Kit (BioFire Defense) according to the manufacturer’s protocol with the following modifications; Roche MagnaLyser was utilized for bead agitation/cell disruption for 15 sec at 3000 rpm, a second cleanup/concentration step was implemented using a 1:1 ratio magnetic beads to eluate. Samples that had sufficiently high DNA content (> 5ng/μL) after amplification were sequenced (n = 4/ anatomical location). 16S rDNA amplification, library creation, and sequencing using Illumina MiSeq was performed at the Forsyth Institute (Cambridge, MA) HOMINGS core. Briefly, libraries were created by amplifying the V1V3 region of 16S rDNA using barcoded 518F (5’- TATGGTAATTCAATTACCGCGGCTGCTGG-3’) and 27R (5’- AGTCAGTCAGCCGAGTTTGATCMTGGCTCAG-3’) primers (Integrated DNA Technologies) that contained Illumina adaptors and cleaned up using Ampure beads (Beckman Coulter Life Sciences). Libraries were pooled, run on a 1% agarose gel, and isolated using a Minelute Gel extraction kit (Qiagen). Sequencing was then performed using a 2x250 V2 cartridge using 518F and 27R primers. An additional indexing primer (5’- CTGAGCCAKGATCAAACTCGGCTGACTGACT-3’) was also used. Sequences were deposited in the NCBI Sequence Read Archive (https://www.ncbi.nlm.nih.gov/Traces/sra/) under biosample accession number SAMN05948837.

### Bioinformatics analyses

Sequencing analysis was performed using MacQIIME [24] (version 1.9.1–20150604) using the pipeline described by the Werner Lab (http://www.wernerlab.org). Sequences were merged and de-multiplexed, which identified 1,591,186 sequences across all samples. 26,351 chimeras were detected and removed from further analysis using USEARCH version 4.2.40 and the gold.fa reference database (http://drive5.com/uchime/uchime_download.html). Using UCLUST [25] at a 97% threshold similarity, *de novo* operational taxonomic units (OTUs) were assigned to each read. A representative sequence for each OTU was then assigned and yielded 16,705 OTUs. Using the Greengenes reference database [26], taxonomy was assigned to generate an OTU table was created followed by creation of a filtered alignment to build a phylogenic tree using FastTree [27]. Rarefactions were then performed in multiples of ten and alpha diversity calculated. The number of observed species was calculated from 50 reads to 40,000 reads. Other indices of alpha diversity were calculated at the 40,000-reads/sample thresholds. These included Chao1, PD_whole tree, Shannon and Simpson reciprocal analysis. Weighted and un-weighted UniFrac [28] indices of beta diversity were estimated. PERMANOVA statistics were performed on both measures, and principal coordinate analysis (PCoA) plots were generated. A Venn diagram was created using Venny (http://bioinfogp.cnb.csic.es/tools/venny/index.html).

### CFU Estimation

Swabs were vortexed for 1 minute, and 1:10, 1:100, 1:1000 serial dilutions were plated on Blood Agar plates (Remel) and placed at 37°C under oxic conditions for 48 hours. Saline solution alone was plated as a negative control. Following a 48 hour incubation, plates were digitally photographed and those with 30 to 300 colonies were counted.

### Fibroblast and Keratinocyte Culture

Primary Normal Human Dermal Fibroblasts (NHDF) and Normal Human Epidermal Keratinocytes (NHEK) were provided by Dr. Robert Christy lab under an approved institutional review board protocol (# H-11-020) by isolation techniques previously described [29–31]. Briefly, tissue harvested during an abdominoplasty underwent a 2 unit/mL dispase (Gibco) digestion, which was used to isolate epidermal versus dermal layers that were processed separately. NHEKs were isolated by mincing epidermal layer that was then filtered, centrifuged, and plated. NHDFs were isolated through mincing and subsequent digestion in 10 mg/mL collagenase (Gibco) in Hank’s Balanced Salt Solution (HBSS, Gibco) with 1% Fetal Bovine Serum (FBS, Gibco) and 1% Antibiotics/Antimycotics (AB/AM, Gibco). NHDFs were then filtered, centrifuged, and plated. NHEKs and NHDFs exhibit expected cobblestone and spindle morphology, respectively, and no signs of contamination. NHDF cells, passage 5 through 7, were cultured in α-Minimally Essential Media (MEM, Gibco) containing 10% FBS and 1% AB/AM at 37°C supplemented with 5% CO_2_. NHEK cells, passage 4 through 6, were cultured in Keratinocyte Media (KM, Promocell) containing all supplements and 1% AB/AM (Gibco) at 37°C supplemented with 5% CO_2_. All cells were grown to 80% confluency and passaged using .25% Trypsin/EDTA solution (Gibco).

### Bacterial Expansion and Bacterial-Treated Media (BTM) Preparation

Swabs were vortexed for 1 minute and subsequently diluted 1:5 in MHBII and placed at 37°C under oxic conditions on a shaker for 24 hours. A 100uL aliquot was added to a 96-well plate to obtain the optical densities (OD) at 600nm for approximate bacterial concentrations. To make BTM, bacterial suspensions were centrifuged for 12 minutes at 4000g and pellets were then suspended in 5mL of either MEM or KM without AB/AM. Following an additional 24-hour incubation, samples were centrifuged for 12 minutes at 4000g, and supernatant was sterilized through 0.22μm syringe filters, confirmed by plating on Blood Agar plates. The pH was determined to be between 7.0 and 7.5 before use with any cells. The bacterial pellet was frozen at -20°C for later culture analysis of the polymicrobial status of BTM cultures. A Pierce BCA Protein Assay (Thermo Fisher) was performed on all BTM, according to manufacturer’s protocol, to quantify protein concentrations.

### Migration Assays

Migration assays were performed similarly to that previously described [32]. Briefly, a solution containing a 1:4 ratio of BTM and corresponding growth media (referred to as MEM-BTM for NHDF assays and KM-BTM for NHEK assays) was utilized. Functional migration assays of NHEKs and NHDFs were performed using Oris Cell Migration Plates (Platypus Technologies). Twenty-Five thousand cells of either NHDF Passage 6 or NHEK Passage 6 were seeded in 100uL in each well and allowed to adhere for four hours. Well inserts were removed and KM-BTM, MEM-BTM or growth media alone were added to triplicate wells, with inserts left in negative control wells. NHDFs and NHEKs were allowed to migrate for up to 24 hours, and cells were then stained with Calcein AM (Thermo Fisher) and imaged using an inverted microscope (Olympus, Inc.). NHDF migration was quantified with a BioTek SynergyMx Plate Reader using the provided plate screen by reading fluorescence (Ex_485_, Em_530_) of only the cells that have migrated inward. NHEK retraction was quantified manually using the area function of ImageJ (National Institutes of Health).

### NHEK-BTM Interaction

Twenty-five thousand NHEKs were also seeded on regular 96-well tissue culture plates and incubated in KM-BTM for 24 hours, followed by fixation with 4% paraformaldehyde (Electron Microscopy Sciences). Cells were blocked and permeablized with 1% Bovine Serum Albumin (BSA, Sigma) and 0.1% Triton-X100 (Fisher) in HBSS. Cells were incubated with either: Caspase-3 (Abcam, Cat. #: ab4051, lot # GR189279-7, 1:300 dilution) or Cytokeratin-14 (Thermo Fisher, Cat #: RB-9020-P, lot #: 9020P1006I, 1:100 dilution) antibodies in HBSS for 2 hours at RT. Cells were then washed, and again blocked with 1% BSA and 0.1% Triton-X100 in HBSS for 1 hour. Fluorescently labeled secondary antibody (ThermoFisher, 1:500 dilution) was then applied and incubated for 1h at RT. Plate(s) were washed, and 0.5ug/mL DAPI (Thermo Fisher) in HBSS solution was applied. Cells were then imaged and the Caspase-3:DAPI ratio was calculated using ImageJ (National Institutes of Health) using the analyze particles feature. In addition to cell imaging, KM-BTM from the experiment described above was collected for ELISA analysis. Replicate mediums were pooled and used undiluted in human Vascular Endothelial Growth Factor (VEGF) ELISA (R&D) according to manufacturer’s protocol.

### Statistics

A power analysis was done to compare 4 samples (AL) via one-way ANOVA with 6 pairwise comparisons, based on pilot CFU counts. Means of 50000 and 20000, and a standard deviation of 15000 were used for sample size calculation, yielding n = 7. For sequencing, pre-established criteria included only those samples with a final DNA concentration over 5ng/μL, yielding n = 4 for each anatomical location (average DNA content of 14.21 ± 1.64 ng/μL). Two-way ANOVA analysis with Tukey’s post-testing was performed at each taxonomic level to determine differences in diversity between anatomic locations. To evaluate if each AL represented distinct a community, PERMANOVA analysis was performed in QIIME. For migration assays, caspase staining, and ELISAs, one-way ANOVA with Tukey’s Post-testing was used to compare anatomical locations while media controls were compared with log-transformation and t-tests assuming unequal variance. Equal variance was found in all parameters, as found by Brown-Forsythe testing. Unless otherwise stated, values are represented as arithmetic mean ± SEM.

## Results

### 16s rDNA sequencing

**Fig 2:**
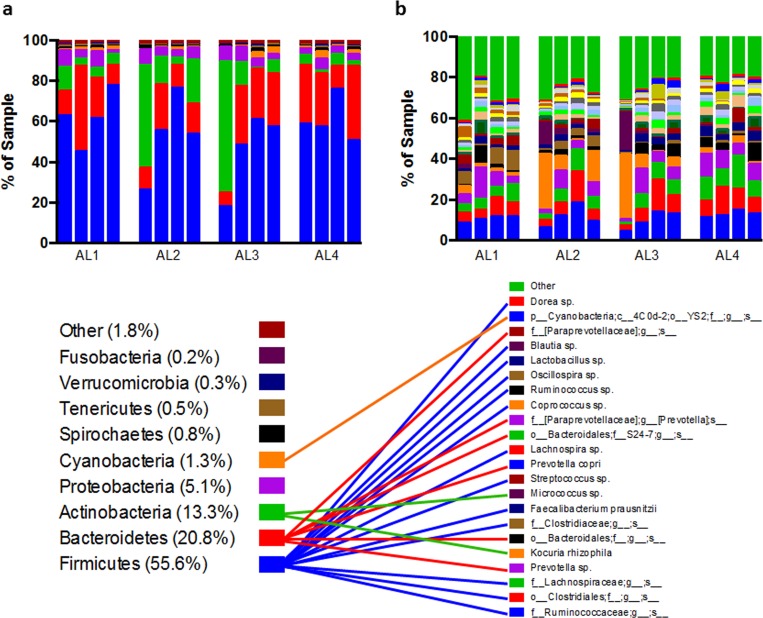
Top phyla and genera grouped by anatomic location. Phyla (a) and species (b) representing, on average, greater than 0.1% or 1% of all sequences, respectively. Percentages represent abundance of phyla averaged across all samples. Lines between keys link taxonomy of each OTU shown. Two samples (from AL2 and AL3) exhibit abnormally high Actinobacteria (green bars in (a)), specifically *Kocuria rhizophila* and an unidentified *Micrococcus* species. Grouped by anatomic location, biological replicates (n = 4/AL) are shown with each bar representing one animal.

While spatial heterogeneity of the microbiome has been documented in humans, the normal porcine microbiome is largely unstudied. We chose to characterize the microbial flora of different anatomical locations previously shown to differ in wound healing studies via 16s rDNA sequencing. We identified a diverse community of both bacteria and archaea that primarily belonged to the *Firmicutes* (55.6 ± 4.0%), *Bacteroidetes* (20.8 ± 2.6%), *Actinobacteria* (13.3 ± 4.6%), and *Proteobacteria* (5.1 ± 0.5%) phyla, among others (Fig 2). At the species level, the most common bacteria included unidentified species of the Ruminococcaceae family (11.3 ± 0.9%), Clostridiales order (8.2 ± 1.0%), and Lachnospiraceae family (7.4 ± 0.9%). Of those species that represented on average greater than 1% of the skin bacteria, three were identified by name: *Kocuria rhizophila* (7.0 ± 2.4%), *Faecalibacterium prausnitzii* (2.8 ± 0.3%), and *Prevotella copri* (2.4 ± 0.5%)

**Fig 3:**
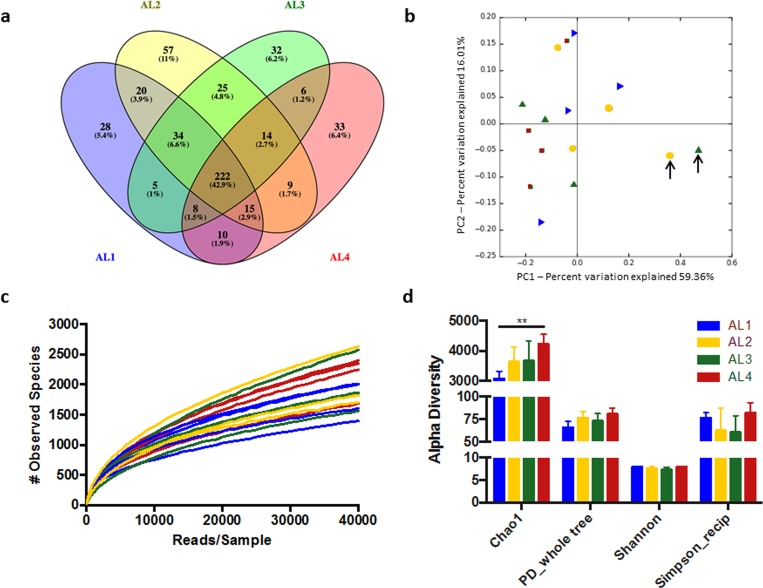
**Species overlap, composition, and alpha diversity by anatomic location** (a) Venn diagram showing the overlap of the 518 identified species between anatomic locations (n = 4/AL). (b) Weighted PCoA Plots wherein each point (n = 16) represents a single swab, reveals the swabs containing abnormally high Actinobacteria, indicated by the arrows. PERMANOVA analysis revealed no significant grouping between anatomic locations (p>0.05). (c) Rarefaction curves for each swab from zero to 40,000 reads per sample showing the number of observed species at each read depth. (d) Chao1 (richness, p = 0.0054), PD_whole tree (diversity, n.s.), Shannon (diversity, n.s.), Simpson (Evenness, n.s.) indices of alpha diversity. AL1 (blue), AL2 (yellow), AL3 (green), AL4 (red). **p<0.01 (2-way ANOVA with Tukey’s Post-testing n = 4/AL).

To give insight into the distribution of specific bacterial species, we examined the 518 unique sequences identified which are listed in S1 Table. All of the 20 bacterial genera identified in chronic wounds (as summarized in Misic, et al [33]) were identified in at least one sample with the exception of the *Morganella* genus. Of the 518 total bacterial species identified, 222 (42.9%) were present in all anatomical locations, while 150 (29.0%) were unique to one particular location (Fig 3A). More species were unique to dorsal locations (i.e., AL1 and 2, 3.9%) than ventral locations (AL3 and 4, 1.2%). For example, the *Finegoldia* genera (0.041 ± 0.04 and 0.009 ± 0.006% in AL1 and AL2, respectively), was only present in dorsal locations. Moreover, the *Oligella* genera were only observed in AL1 (0.033 ± 0.003%) while the *Janthinobacterium* genera was only observed in low abundance one sample in AL2. Other known pathogenic genera identified by Misic et al. such as *Pseudomonas* spp., *Porphyromonas* spp., and *Staphylococcus* spp. were observed in all locations, but in relatively low abundance.

Differences in wound healing in the dorso-ventral and cranio-caudal anatomical axes may be influenced by differences in the local microbiome. Thus, we separated sequencing data on those axes for quantitative analysis of species differences between anatomic locations as delineated in Table 1 and Fig 1. Of note, there were significantly less *Streptococcus* species in AL3 compared to all other locations. Significant site-specific differences in *Faecalibacterium prausnitzii*, *Kocuria rhizophila*, and in *Lactobacillus* and *Micrococcus* species were also observed, even following elimination of the AL2 and AL3 swabs from the pig with high Actinobacteria (data not shown). Examination of differences between Cranial (AL1, AL3) and Caudal (AL2, AL4) swabs revealed a significant elevation of unidentified species of the Clostridiales order (p = 0.04), the Lachnospiraceae family (p<0.0001), and the Ruminococcaceae family (p<0.0001).

**Table 1:** Species level differences by anatomic region.

Class	Order	Family	Name (genus, species)	% Dorsal	% Ventral	p-value	% Cranial	% Caudal	p-value
Bacteroidia	Bacteroidales	unnamed	unnamed	2.42	4.12	0.0002	3.35	3.19	ns
Bacilli	Lactobacillales	Lactobacillaceae	Lactobacillus sp.	2.15	0.62	0.0021	1.80	0.97	ns
Clostridia	Clostridiales	unnamed	unnamed	7.11	9.22	< 0.0001	7.50	8.83	0.0446
		Clostridiaceae	unnamed	4.91	0.85	< 0.0001	3.48	2.28	ns
		Lachnospiraceae	unnamed	6.21	8.50	< 0.0001	5.95	8.76	< 0.0001
		Ruminococcaceae	Faecalibacterium prausnitzii	1.98	3.57	0.0009	2.52	3.03	ns
			unnamed	10.99	11.54	ns	10.32	12.21	< 0.0001

Species-level analysis of differences comparing Dorsal (AL1 & AL2) v. Ventral (AL3 & AL4) and Cranial (AL1 & AL3) v. Caudal (AL2 & AL4) swab sites. Only species with significant differences (2-way ANOVA with Tukey’s Post-testing, n = 4/AL, p<0.05) are shown.

While species-level differences due to anatomical location are apparent, we sought to identify if the microbiome from these locations represented distinct microbial communities, and if they differed in diversity. Weighted principle coordinate analysis (PCoA) (Fig 3B**)** revealed the distinct community represented by the swabs with high amounts of Actinobacteria; however, this observation diminished in un-weighted analysis (S1 Fig). Moreover, no significant grouping by anatomic location was seen as revealed by PERMANOVA analysis of weighted or un-weighted beta diversity scores. Rarefaction analysis (Fig 3C) and alpha diversity calculations (Fig 3D) showed a significant increase in Chao1 (richness) index from AL1 to AL4 (p = 0.0054). This trend was lso evident in PD_whole tree analysis (diversity) but not in Shannon (diversity) or reciprocal Simpson (evenness) scores.

### Bacterial Treated Media (BTM) Preparation

**Fig 4:**
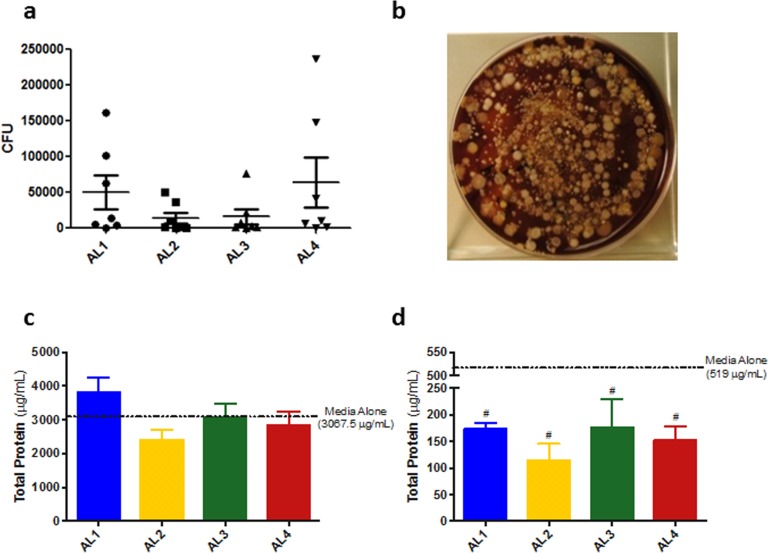
Bacterial load and culture. (a) Number of colony forming units (CFU) for each anatomic location (n = 7/AL). One-way ANOVA revealed no significant differences between anatomic locations. (b) Representative 1-10-fold dilution of CFU plates is displayed showing a diverse polymicrobial community. Protein content is similar between anatomic locations for MEM-BTM (c) and KM-BTM (d). KM-BTM had a significant reduction in protein content compared to KM alone (p = 0.004) however MEM-BTM did not.

The amount of bacteria from different anatomical locations, and their expansion in media used for *in vitro* studies was examined. Initial CFU counts from all swabs were determined, and no significant differences were observed between anatomical locations (Fig 4A). Visual inspection of the plates revealed a diverse poly-microbial population that was easily cultured (Fig 4B**)**. Bacterial concentrations as approximated by OD_600_ values did not differ after expansion in Mueller-Hinton Broth II (MHBII), α-Minimally Essential Media (MEM) or Keratinocyte media (KM) (S2 Fig). Bacterially treated MEM (MEM-BTM) (Fig 3C) and bacterially treated KM (KM-BTM) (Fig 4D) had similar protein concentrations from all anatomical locations. However, KM-BTM from all locations had a lower protein content compared to KM alone (p<0.004), indicating significant protein catabolism by bacteria during incubation (Fig 4D). Culturing of unfiltered KM-BTM and MEM-BTM also revealed a poly-microbial community (S2 Fig), with similar bacterial load.

### The effect of bacterial supernatant on human fibroblast and keratinocyte cell migration

**Fig 5:**
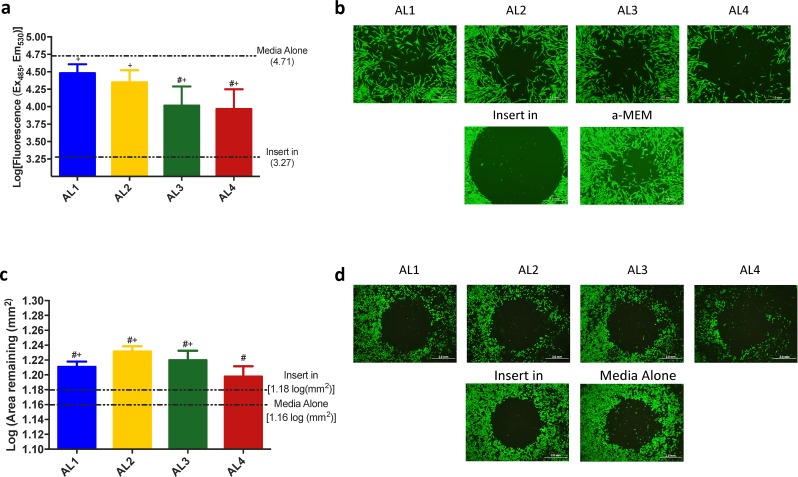
The effect of MEM-BTM on Fibroblast and Keratinocyte migration. (a) Fibroblasts seeded on cell migration plates were allowed to migrate overnight. Fibroblasts incubated with BTM from all locations migrated more than the negative control (p<0.04) however migration was impeded in BTM from ventral swabs (AL3, p = 0.04 & AL4, p = 0.04) compared to a-MEM control (two-tailed unpaired t-test, n = 7/AL). #, p<0.05 v. Media control; +, p<0.05 v. “Insert in” Control. (b) Respective 4x images of each location are shown and scale bars represent 1.0 mm. (c) Keratinocytes seeded on cell migration plates were allowed to migrate overnight. NHEKs incubated with BTM from all locations exhibited retraction compared to media control (p<0.05), however AL1 (p = 0.004), AL2 (p = 0.0003), and AL3 (p = 0.02) also exhibited significant retraction compared to negative control (two tailed unpaired t-test, n = 7/AL). #, p<0.05 v. Media control; +, p<0.05 v. Insert In Control. (d) Respective 2x images of each location are shown and scale bars represent 2.0 mm. Images are a representation of two independent experiments.

We wanted to then see if proteins released from bacteria influenced *in vitro* assays of wound healing using human cells. We applied bacterial-released proteins to NHDF and NHEK cells grown in a cell migration plate. As shown in Fig 5, MEM-BTM from all anatomical locations supported NHDF migration compared to the negative control, indicating that there was no adverse effect on cell viability. While all BTM showed lower fluorescence reading compared to normal MEM, only MEM-BTM prepared from ventral sites inhibited migration of NHDFs compared to MEM alone (p = 0.044 and p = 0.039 for AL3 and AL4, respectively). NHEKs in both positive and negative control groups did not display sufficient migration to utilize the fluorescence reading ability of cell migration plates. Moreover, KM-BTM from all locations not only exhibited decreased cell densities within the insert, but also increased the remaining area without NHEKs compared to KM control (Fig 5).

### The influence of bacterial supernatant on NHEKs

**Fig 6:**
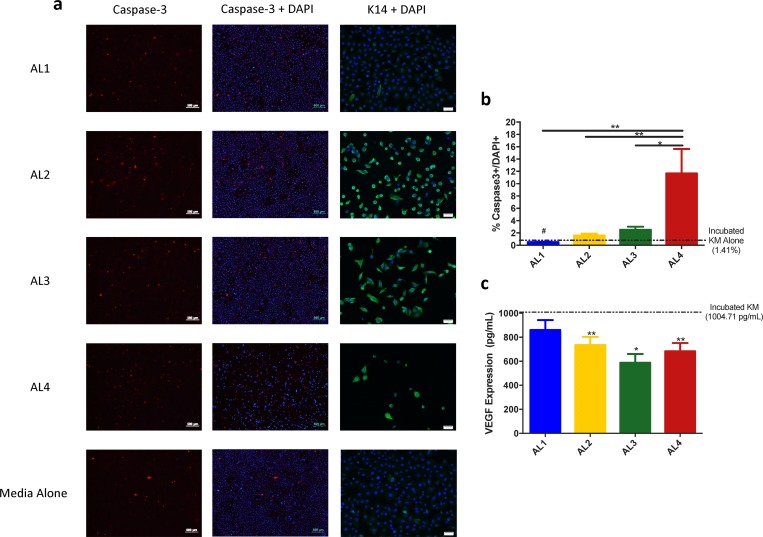
The effect of KM-BTM on Keratinocyte viability, differentiation, and VEGF release. (a and b) NHEKs were treated with KM-BTM, fixed, and stained with caspase-3. The percentage of cells expressing caspase-3 is higher in NHEKs treated with BTM from AL4 compared to other locations (p<0.02) and lower in AL1 (p = 0.0003) compared to incubated KM. (a, right column) Cytokeratin-14 expression was maintained when incubated in BTM from AL2-4 but not in AL1 or KM alone. For caspase-3 stain, 4x images are shown and scale bars represent 500 μm. For K-14 stain, 10x images are shown and scale bar represents 50 μm. (c) VEGF protein levels in cell supernatant were decreased following incubation in BTM from all locations (p<0.01) except for AL1 compared to incubated keratinocyte media (two tailed unpaired t-test, n = 7/AL).

The previous finding indicated that BTM negatively affected the viability of NHEKs. To explore the mechanistic basis behind the reduction in NHEK density, we examined the expression of the apoptosis marker Caspase 3, and the active keratinocyte marker Keratin14 on NHEKs. As shown in Fig 6A (quantified in Fig 6B), KM-BTM from AL4 increased Caspase-3+ cells compared to the other three sites (p = 0.0022), while that from AL1 decreased Caspase-3+ cells compared to KM (p = 0.0003). This difference is due, in part, to the reduction in the total amount of NHEKs grown in BTM from AL4, suggesting other mechanisms are also involved. NHEKs incubated in KM-BTM from AL2-4 also continued to express the basal-keratinocyte marker cytokeratin-14, while cells incubated with media from AL1 or KM alone reached confluence and lost this expression (Fig 6A).

In addition to examining NHEK expression after BTM incubation, we also performed ELISAs on the media supernatant for growth factor analysis. Compared to KM-incubated NHEKs (1004.714 pg/mL), a significant decrease in VEGF release was seen in NHEKs incubated with KM-BTM from all location except AL1 (Fig 6C). These differences are not attributable to any amount of VEGF present in KM (which was undetectable) or un-incubated KM-BTM (7.1 pg/mL on average), but rather indicate that KM-BTM from AL2-4 inhibits VEGF release from NHEKs. Analysis of Keratinocyte Growth Factor (KGF) did not reveal any appreciable amount of KGF in any group, including the media control.

## Discussion

Recent advances in sequencing technology have revealed vast amounts of information about the human skin microbiome, yet animal models of these complex communities are lacking. Although studies of the microbiome’s effect on cutaneous wound healing have been performed in rodents [7] and dogs [34], such studies have not been performed in pigs. This is despite the pig being widely regarded as the best clinical surrogate for wound healing studies due to similarities in architecture (e.g., hair follicle density, epidermal thickness, etc.) and the mechanisms of wound healing (i.e., contraction vs. epithelialization) to the human skin [16–18]. In the current study we present evidence of similarities and differences in the cutaneous microbiomes from swine to humans that may be leveraged to study different variables such as age, anatomical location, or disease. Moreover, given that proteins released from bacterial populations on pig skin affect human cells, the pig is well suited for further studies examining the role of natural flora on homeostasis and wound healing.

### The porcine cutaneous microbiome and wound healing

While the human skin bacteriome harbors the same top four phyla as reported in this study, their relative abundances are slightly altered [3]. At the species level, we identified several unnamed organisms representing relatively high abundances on the pig skin. These included species of the *Lactobacillus*, *Ruminococcus*, *Streptococcus*, and *Micrococcus* genera. The most abundant species were unnamed organisms of the *Ruminococcaceae* (11.4%), *Lachnospiraceae* (7.4%) families and *Clostridiales* (8.2%) order. This is in contrast to the high abundance of *Propionibacterium*, *Corynebacterium*, and *Staphylococcus* commonly found on human skin [3], which only represented 0.0045 ± 0.006% of sequences, 0.33 ± 0.3%, and 0.57 ± 0.5% of the pig skin bacteriome, respectively. While *Kocuria rhizophila*, the fourth most abundant species in our samples, has been shown to cause systemic infection, its role in cutaneous wound healing is unclear [35].

Interestingly, a large proportion of species found on pig skin are also constituents of the pig gut microbiome. This could, in part, be due to overlap in community composition, or the fact that that the pig lifestyle promulgates interactions between the fecal bacteriome and the skin. For example, *F*. *prausnitzii*, which represents 2.8% of the pig skin bacteriome, makes up 6.57% of the pig fecal microbiome [36] and 9.8% of the human gut microbiome [37]. Studies of the human gut microbiome have shown that intestinal *F*. *prausnitzii* influences atopic dermatitis through immunomodulation of the T_H_2 response to inflammation [38]. In our animals, we observed significantly higher amounts of *F*. *prausnitzii* in AL4 (4.1 ± 0.5% of sequences) compared to AL1 (2.0 ± 0.5% of sequences) and AL2 (2.0 ± 0.4% of sequences). While this anatomic location is in closer proximity to fecal matter it is not often the location of choice in wound healing studies due to difficulty in applying dressings. It is not known whether this bacterium, or other gut flora, have direct effects on cutaneous wound healing when present on the skin, although modulation of the gut flora indirectly affects cutaneous wound healing [39].

### Many variables affect the cutaneous microbiome

To our knowledge, this is the first study that examines cutaneous bacterial populations in swine from multiple anatomical locations. One previous study by Swe et al. focused on disease (i.e., scabies)-driven changes in the microbiome of the inner surface of the ear [23]. They did, however, find in healthy swine that the most common genera were *Streptococcus* and *Lactobacillus*, which were also represented in 2.41% and 1.65% of the species we identified in the current study. These authors also found that as the pigs aged 21 weeks (starting at about 3 weeks of age), there was a significant reduction in microbial diversity. This is at odds with what is seen in humans, which has been shown to be an increase in diversity after infancy [40, 41]. Additional factors such as the rigorously maintained housing facilities could be a possible explanation for this discrepancy. Regardless, direct comparisons of these two pig populations (as well as those to humans) are difficult to compare due to vast number of variables to include age and species/breed. As such, direct comparisons within studies are much easier to interpret and, while previously known in humans [2], differences in the microbiome at different anatomical locations in swine have not been studied.

To this end, we also observed a decreased Chao1 index in AL1 compared to AL4, indicating decreased species richness. While we hypothesized that the relatively moist environment of the ventral swabs would provide contrasting microbial communities with the swabs of the dorsum (AL1 and AL2), perhaps more extreme locations could have been chosen (i.e., hoof, snout, jowls, etc.). Interestingly, however, in two individual swabs we observed an abnormally high abundance of Actinobacteria, including *K*. *rhizophila* and of an unnamed *Micrococcus* species. This resulted in distinct communities found by weighted PCoA analysis, but not by unweighted analysis indicating abnormal bacterial abundance but not presence. Whether these changes might represent a dysbiosis is a subject for future investigations of swine health. In light of the observation that we did not find high amounts bacterial genera commonly found in chronic wounds, these results could indicate an altered species-level profile compared to humans.

One aspect of anatomical variability this study does not address is that of tissue depth. A previous study has eloquently showed that there are several subepidermal niches of bacteria under normal conditions [4]. Indeed, different niches such as sebaceous or sweat glands are likely not represented in the data presented herein. The fact that these pigs were unshaven, however, does indicate that this population includes bacteria associated with hair. From a wound healing perspective, bacteria that colonize the most superficial aspect of a wound often do not result in clinical infection until the bacteria has reached deeper areas of tissue. In this regard, homogenizing tissue for analysis of the bacteria may result in distinct populations.

### Effects on human cells

We also found that supernatant from pig bacteria cultured *in vitro* had anatomic location-specific effects on human keratinocytes (NHEKs) and in fibroblasts (NHDFs). Specifically, BTM from ventral locations significantly reduced NHDF migration compared to media alone. These results indicate potential implications for scarring, as it is known that bacterial infections can cause fibroblast proliferation [42, 43], a hallmark of hypertrophic scarring [44]. Previous studies in pigs have shown that cranial wounds exhibit slower re-epithelization and higher contraction compared to caudal sites [21] although this could have been a result of differences in wound depth [45]. Regardless, the role of normal bacterial flora on fibroblast proliferation and collagen deposition remains to be elucidated. The fact that proteins from bacterial populations on pig skin affect human cells suggests that swine could be used to assess the interplay between the microbiome and topical treatments, injuries, or infections.

As keratinocytes likely have greater exposure to bacteria *in vivo* due to their location on the skin, we hypothesized that NHEKs would display enhanced sensitivity to KM-BTM. In line with this, bacterial supernatant from AL1 and AL4 consistently showed contrasting effects on NHEKs. KM-BTM from only AL1 produced statistically similar VEGF release, decreased caspase-3 staining and an identical K-14 staining pattern compared to KM alone. This suggests KM-BTM from AL1 (and normal KM) supported NHEK viability and proliferation, with subsequent differentiation once confluency was reached. On the contrary, NHEKs incubated in KM-BTM from AL4 exhibited strong K-14 staining, decreased VEGF release, and drastically decreased cell viability. These results suggest the possibility of detrimental effects on wound healing (i.e. delayed reepithelialization) dependent on anatomical location, which warrants further investigation.

### Implications

Taken together, these findings could have not only clinical implications, but also on pig models that investigate wound healing. For example, such studies commonly utilize multiple wounds on a single pig in order to reduce cost and resource investment. As previously mentioned, anatomic location can effect wound healing, which can confound results if not properly controlled. The mechanisms of this phenomenon are not fully understood, however they may be a result of differences in skin thickness, especially of the subcutaneous layer, and in hair follicle density [45]. The results presented herein suggest that the cutaneous bacteriome may also be influencing location-dependent differences in wound healing. While the effect of pathogenic bacteria (including biofilms) on human cell migration has been studied previously [46], to our knowledge this is the first study that examines changes in human cell migration due to normal bacterial flora.

We acknowledge that our study has several limitations. First, we only examined healthy female swine of a young age, with similar environmental cues and genetic makeup. Our choice of relatively young (3 month old) pigs reflects the usual age for wound healing studies in swine, due to the logistical constraints of handling larger animals [16]. However, it is known that age affects not only wound healing in swine [22], but also the microbiome of humans [47]. Similarly, while Yorkshire pigs are often used for wound healing studies, other species have been incorporated depending on the research question, such as Red Duroc pigs for studying hypertrophic scarring [48]. While several studies examine the differences between molecular signaling and host fibroblasts between these two species [49, 50], the influence of the microbiome could be an exciting avenue of future research. [3]. Second, although included in sequencing analysis, obligate anaerobic bacteria (which do influence wound healing) were not cultured and were thus excluded from *in vitro* analysis. Third, we did not perform 16S rDNA sequencing on BTM preparations, and due to species selection, proteins in the BTM may be an inferential reflection of the bacteriome. Future studies could investigate these observations in addition to a temporal shift that may occur as these animals age and respond to stress or injury.

## Conclusions

In summary, this study characterizes the pig skin bacteriome beyond the inner ear, setting the stage for a number of studies. Specifically, future work could focus on the impact of topical treatments, injuries, and infections on the porcine microbiome and cutaneous wound healing. We found that although humans and pigs share similar phyla on the skin, species-level differences do exist. However, further studies are needed to investigate their impact on wound healing. Likewise, we found that the media conditioned by normal flora bacteria from porcine skin influenced human fibroblast migration as well as human keratinocyte viability, and growth factor release. As a result, we suggest that the pig could serve as a suitable model in future studies examining the impact of the microbiome on various skin disorders and wounds.

## Supporting Information

S1 Fig Unweighted PCoA Plot. Arrows identify swabs that contain abnormally high Actinobacteria abundance that result in distinct communities in weighted analysis (Fig 3B). PERMANOVA analysis revealed no significant grouping between Anatomic Locations. Each point (n = 16) represents a single swab and is colored based on anatomic location: AL1 (blue), AL2 (yellow), AL3 (green), AL4 (red).
(TIF)

S2 Fig Bacterial culture and BTM preparation (a) Bacterial growth following 24 hours of incubation in MHBII broth (n = 7/AL). Bacterial pellets were then split and resuspended in either a-MEM or KM and incubated for an additional 24 hours to make BTM. (n = 7/AL/media) (b) MEM-BTM showed no significant differences between anatomical locations in bacterial density following incubation. (c) A representative image of colonies following incubation in MEM-BTM that retained a poly-microbial population. (d) KM-BTM also exhibited no significant differences in bacterial density between anatomical location and a diverse population following incubation in KM. (e) A representative image of colonies following incubation in MEM-BTM that retained a poly-microbial population. Images of 1–10,000 dilutions are shown.
(TIF)

S1 Table % of identified species. Each of the 518 identified species in relative abundance from each anatomical location and the average is shown to the lowest identified classification.
(DOCX)

S2 Table Species level differences by anatomic location. Species-level analysis of differences comparing all swab sites. Only species with significant differences (2-way ANOVA with Tukey’s Post-testing, n = 4/AL, p<0.05) are shown.
(DOCX)

## References

[1] Naik S, Bouladoux N, Wilhelm C, Molloy MJ, Salcedo R, Kastenmuller W, et al Compartmentalized Control of Skin Immunity by Resident Commensals. Science. 2012;337(6098). 10.1126/science.1225152 PMC3513834 22837383

[2] Grice EA, Segre JA. The skin microbiome. Nature Reviews Microbiology. 2011;9(4). 10.1038/nrmicro2537 PMC3535073 21407241

[3] Grice EA, Kong HH, Conlan S, Deming CB, Davis J, Young AC, et al Topographical and Temporal Diversity of the Human Skin Microbiome. Science. 2009;324(5931). 10.1126/science.1171700 PMC2805064 19478181

[4] Nakatsuji T, Chiang H-I, Jiang SB, Nagarajan H, Zengler K, Gallo RL. The Microbiome Extends to Subepidermal Compartments of Normal Skin. Nature Communications. 2013;4(1431). 10.1038/ncomms2441 PMC3655727 23385576

[5] Gallo RL, Nakatsuji T. Microbial Symbiosis with the Innate Immune Defense System of the Skin. Journal of Investigative Dermatology. 2011;131. 10.1038/jid.2011.182 PMC3174284 21697881

[6] Kong HH, Oh J, Deming C, Conlan S, Grice EA, Beatson MA, et al Temporal shifts in the skin microbiome associated with disease flares and treatment in children with atopic dermatitis. Genome Research. 2012;22. 10.1101/gr.131029.111 PMC3337431 22310478

[7] Grice EA, Snitkin ES, Yockey LJ, Bermudez DM, Programc NCS, Liechty KW, et al Longitudinal shift in diabetic wound microbiota correlates with prolonged skin defense response. PNAS. 2010;107(33). 10.1073/pnas.1004204107 PMC2930465 20668241

[8] Singer AJ, Dagum AB. Current management of acute cutaneous wounds. N Engl J Med. 2008;359(10). 10.1056/NEJMra0707253 18768947

[9] Lai Y, Nardo AD, Nakatsuji T, Leichtle A, Yang Y, Cogen AL, et al Commensal bacteria regulate Toll-like receptor 3– dependent inflammation after skin injury. Nature Medicine. 2009;15(12). 10.1038/nm.2062 PMC2880863 19966777

[10] Lai Y, Cogen AL, Radek KA, Park HJ, MacLeod DT, Leichtle A, et al Activation of TLR2 by a Small Molecule Produced by Staphylococcus epidermidis Increases Antimicrobial Defense against Bacterial Skin Infections. Journal of Investigative Dermatology. 2010;130. 10.1038/jid.2010.123 PMC2922455 20463690

[11] Laborel-Préneron E, Bianchi P, Boralevi F, Lehours P, Fraysse F, Morice-Picard F, et al Effects of the Staphylococcus aureus and Staphylococcus epidermidis Secretomes Isolated from the Skin Microbiota of Atopic Children on CD4+ T Cell Activation. PLOS One. 2015;10(10). 10.1371/journal.pone.0141067 PMC4624846 26510097

[12] Canesso MCC, Vieira AlT, Castro TBR, Schirmer BgGA, Cisalpino D, Martins FS, et al Skin Wound Healing Is Accelerated and Scarless in the Absence of Commensal Microbiota. Journal of Immunology. 2014;193. 10.4049/jimmunol.1400625 25326026

[13] Zeeuwen PL, Boekhorst J, Bogaard EHvd, Koning HDd, Kerkhof PMvd, Saulnier DM, et al Microbiome dynamics of human epidermis following skin barrier disruption. Genome Biology. 2012;13(R101). 10.1186/gb-2012-13-11-r101 PMC3580493 23153041

[14] Zhang M, Jiang Z, Li D, Jiang D, Wu Y, Ren H, et al Oral Antibiotic Treatment Induces Skin Microbiota Dysbiosis and Influences Wound Healing. Microbial Ecology. 2015;69. 10.1007/s00248-014-0504-4 25301498

[15] Takao K, Miyakawa T. Genomic responses in mouse models greatly mimic human inflammatory diseases. Proc Natl Acad Sci U S A. 2015;112(4). 10.1073/pnas.1401965111 PMC4313832 25092317

[16] Singer A, McClain S. A porcine burn model. Methods Molecular Medicine. 2003;78. 10.1385/1-59259-332-1:107 12825265

[17] Sullivan T, Eaglstein W, Davis S, Mertz P. The pig as a model for human wound healing. Wound Repair and Regeneration. 2001;9. 10.1046/j.1524-475x.2001.00066.x 11350644

[18] Ramos M, Gragnani A, Ferreira L. Is there an ideal animal model to study hypertrophic scarring? Journal of Burn Care Research. 2008;29. 10.1097/BCR.0b013e3181667557 18354295

[19] Abdullahi A, Amini-Nik S, Jeschke MG. Animal models in burn research. Cell Mol Life Sci. 2014;71(17). 10.1007/s00018-014-1612-5 PMC4134422 24714880

[20] Seaton M, Hocking A, Gibran NS. Porcine models of cutaneous wound healing. ILAR J. 2015;56(1). 10.1093/ilar/ilv016 25991704

[21] Wang X-Q, Liu P-Y, Kempf M, Cuttle L, Chang AH-E, Wong M, et al Burn healing is dependent on burn site: A quantitative analysis from a porcine burn model. Burns. 2009;35(2). 10.1016/j.burns.2008.05.030 18845398

[22] Yao F, Visovatti S, Johnson CS, Chen M, Slama J, Wenger A, et al Age and growth factors in porcine full-thickness wound healing. Wound Repair Regen. 2001;9(5). 10.1046/j.1524-475x.2001.00371.x 11896980

[23] Swe PM, Zakrzewski M, Kelly A, Krause L, Fischer K. Scabies Mites Alter the Skin Microbiome and Promote Growth of Opportunistic Pathogens in a Porcine Model. PLOS Neglected Tropical Diseaes. 2014;8(5). 10.1371/journal.pntd.0002897 PMC4038468 24875186

[24] Caporaso JG, Kuczynski J, Stombaugh J, Bittinger K, Bushman FD, Costello EK, et al QIIME allows analysis of high-throughput community sequencing data. Nat Methods. 2010;7(5). 10.1038/nmeth.f.303 PMC3156573 20383131

[25] Edgar R. Search and clustering orders of magnitude faster than BLAST. Bioinformatics. 2010;26(19). 10.1093/bioinformatics/btq461 20709691

[26] McDonald D, Price MN, Goodrich J, Nawrocki EP, DeSantis TZ, Probst A, et al An improved Greengenes taxonomy with explicit ranks for ecological and evolutionary analyses of bacteria and archaea. ISME J. 2012;6(3). 10.1038/ismej.2011.139 PMC3280142 22134646

[27] Price MN, Dehal PS, Arkin AP. FastTree 2—approximately maximum-likelihood trees for large alignments. PLoS One. 2010;5(3). 10.1371/journal.pone.0009490 PMC2835736 20224823

[28] Lozupone C, Knight R. UniFrac: a new phylogenetic method for comparing microbial communities. Appl Environ Microbiol. 2005;71(12). 10.1128/AEM.71.12.8228-8235.2005 PMC1317376 16332807

[29] Hybbinette S, Bostrom M, Lindberg K. Enzymatic dissociation of keratinocytes from human skin biopsies for in vitro cell propagation. Exp Dermatol. 1999;8(1). 10.1111/j.1600-0625.1999.tb00345.x 10206719

[30] Normand J, Karasek MA. A method for the isolation and serial propagation of keratinocytes, endothelial cells, and fibroblasts from a single punch biopsy of human skin. In Vitro Cell Dev Biol Anim. 1995;31(6). 10.1007/BF02634257 8589888

[31] Shrivastava HY, Ravikumar T, Shanmugasundaram N, Babu M, Unni Nair B. Cytotoxicity studies of chromium(III) complexes on human dermal fibroblasts. Free Radic Biol Med. 2005;38(1). 10.1016/j.freeradbiomed.2004.09.029 15589372

[32] Ward CL, Jr CJS, Pollot BE, Romano DR, Hardy SK, Becerra SC, et al Soluble factors from biofilms of wound pathogens modulate human bone marrow-derived stromal cell differentiation, migration, angiogenesis, and cytokine secretion. BMC Microbiology. 2015;15(75). 10.1186/s12866-015-0412-x PMC4381664 25886581

[33] Misic AM, Gardner SE, Grice EA. The Wound Microbiome: Modern Approaches to Examining the Role of Microorganisms in Impaired Chronic Wound Healing. Adv Wound Care (New Rochelle). 2014;3(7). 10.1089/wound.2012.0397 PMC4086514 25032070

[34] Bradley CW, Morris DO, Rankin SC, Cain CL, Misic AM, Houser T, et al Longitudinal Evaluation of the Skin Microbiome and Association with Microenvironment and Treatment in Canine Atopic Dermatitis. J Invest Dermatol. 2016. 10.1016/j.jid.2016.01.023 PMC4877200 26854488

[35] Moissenet D, Becker K, Merens A, Ferroni A, Dubern B, Vu-Thien H. Persistent bloodstream infection with Kocuria rhizophila related to a damaged central catheter. J Clin Microbiol. 2012;50(4). 10.1128/JCM.06038-11 PMC3318534 22259211

[36] Magistrelli D, Zanchi R, Malagutti L, Galassi G, Canzi E, Rosi F. Effects of Cocoa Husk Feeding on the Composition of Swine Intestinal Microbiota. J Agric Food Chem. 2016;64(10). 10.1021/acs.jafc.5b05732 26877143

[37] Kolmeder CA, Salojarvi J, Ritari J, de Been M, Raes J, Falony G, et al Faecal Metaproteomic Analysis Reveals a Personalized and Stable Functional Microbiome and Limited Effects of a Probiotic Intervention in Adults. PLoS One. 2016;11(4). 10.1371/journal.pone.0153294 PMC4829149 27070903

[38] Song H, Yoo Y, Hwang J, Na YC, Kim HS. Faecalibacterium prausnitzii subspecies-level dysbiosis in the human gut microbiome underlying atopic dermatitis. J Allergy Clin Immunol. 2016;137(3). 10.1016/j.jaci.2015.08.021 26431583

[39] Poutahidis T, Kearney SM, Levkovich T, Qi P, Varian BJ, Lakritz JR, et al Microbial symbionts accelerate wound healing via the neuropeptide hormone oxytocin. PLoS One. 2013;8(10). 10.1371/journal.pone.0078898 PMC3813596 24205344

[40] SanMiguel A, Grice EA. Interactions between host factors and the skin microbiome. Cell Mol Life Sci. 2015;72(8). 10.1007/s00018-014-1812-z PMC4376244 25548803

[41] Capone KA, Dowd SE, Stamatas GN, Nikolovski J. Diversity of the human skin microbiome early in life. J Invest Dermatol. 2011;131(10). 10.1038/jid.2011.168 PMC3182836 21697884

[42] Jones SG, Edwards R, Thomas DW. Inflammation and wound healing: the role of bacteria in the immuno-regulation of wound healing. Int J Low Extrem Wounds. 2004;3(4). 10.1177/1534734604271810 15866816

[43] Stephens P, Wall IB, Wilson MJ, Hill KE, Davies CE, Hill CM, et al Anaerobic cocci populating the deep tissues of chronic wounds impair cellular wound healing responses in vitro. Br J Dermatol. 2003;148(3). 10.1046/j.1365-2133.2003.05232.x 12653737

[44] Wang J, Dodd C, Shankowsky HA, Scott PG, Tredget EE, Wound Healing Research G. Deep dermal fibroblasts contribute to hypertrophic scarring. Lab Invest. 2008;88(12). 10.1038/labinvest.2008.101 18955978

[45] Singer AJ, Toussaint J, Chung WT, Thode HC, McClain S, Raut V. Effects of burn location and investigator on burn depth in a porcine model. Burns. 2016;42(1). 10.1016/j.burns.2015.09.016 26507518

[46] Jeffery Marano R, Jane Wallace H, Wijeratne D, William Fear M, San Wong H, O'Handley R. Secreted biofilm factors adversely affect cellular wound healing responses in vitro. Sci Rep. 2015;5. 10.1038/srep13296 PMC5388174 26278131

[47] Oh J, Conlan S, Polley EC, Segre JA, Kong HH. Shifts in human skin and nares microbiota of healthy children and adults. Genome Med. 2012;4(10). 10.1186/gm378 PMC3580446 23050952

[48] Gallant CL, Olson ME, Hart DA. Molecular, histologic, and gross phenotype of skin wound healing in red Duroc pigs reveals an abnormal healing phenotype of hypercontracted, hyperpigmented scarring. Wound Repair Regen. 2004;12(3). 10.1111/j.1067-1927.2004.012311.x 15225209

[49] Gallant-Behm CL, Reno C, Tsao H, Hart DA. Genetic involvement in skin wound healing and scarring in domestic pigs: assessment of molecular expression patterns in (Yorkshire x Red Duroc) x Yorkshire backcross animals. J Invest Dermatol. 2007;127(1). 10.1038/sj.jid.5700482 16858423

[50] de Hemptinne I, Gallant-Behm CL, Noack CL, Parreno J, Hart DA. Dermal fibroblasts from red Duroc and Yorkshire pigs exhibit intrinsic differences in the contraction of collagen gels. Wound Repair Regen. 2008;16(1). 10.1111/j.1524-475X.2007.00340.x 18211585

