# Supplementary figures and images for: Initial Characterization of the Pig Skin Bacteriome and Its Effect on In Vitro Models of Wound Healing
Source: PLoS One. 2016 Nov 8;11(11):e0166176. doi: 10.1371/journal.pone.0166176 (PMC5100914; doi:10.1371/journal.pone.0166176)

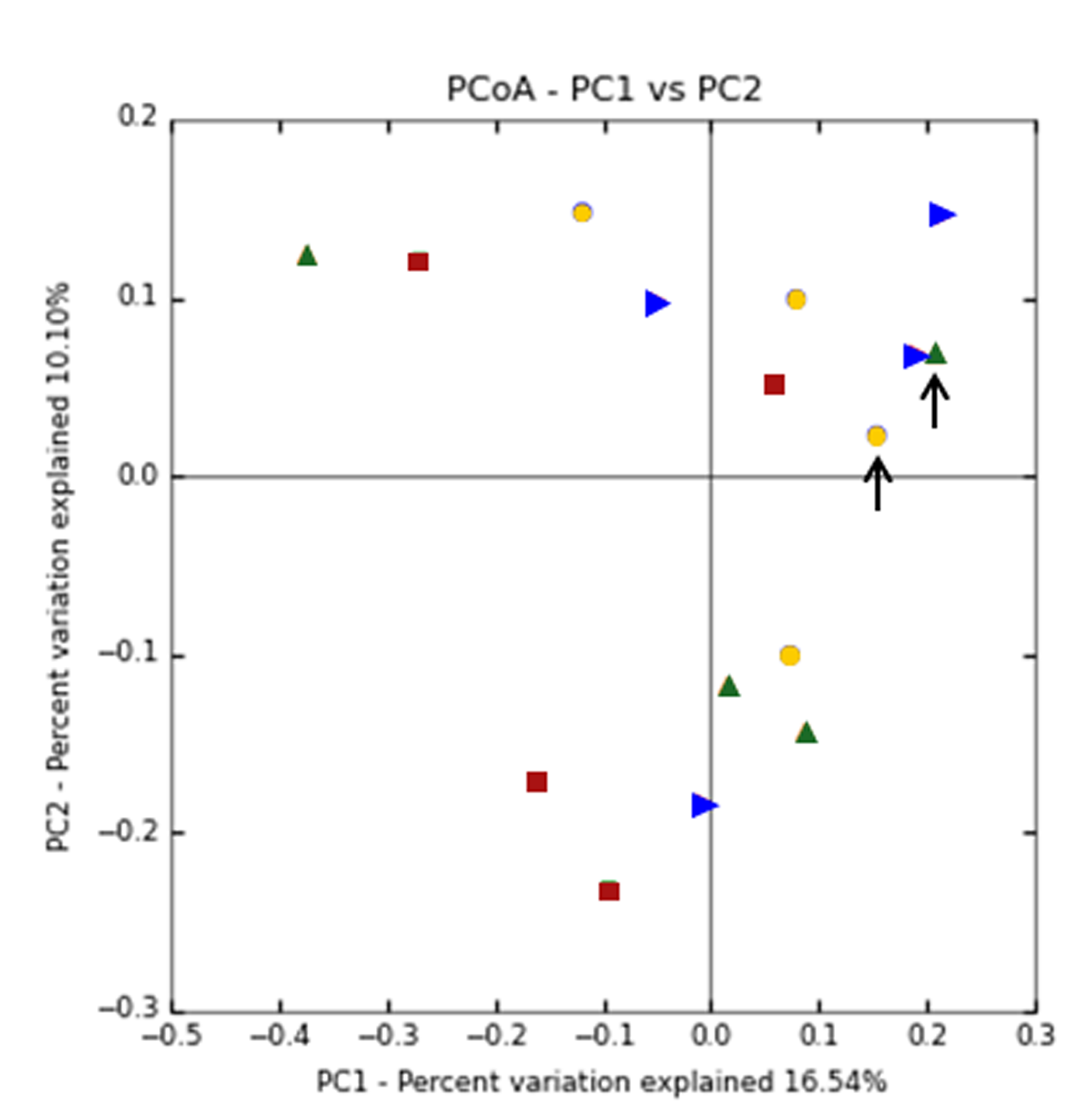

Supplement: S1 Fig — Arrows identify swabs that contain abnormally high Actinobacteria abundance that result in distinct communities in weighted analysis (Fig 3B). PERMANOVA analysis revealed no significant grouping between Anatomic Locations. Each point (n = 16) represents a single swab and is colored based on anatomic location: AL1 (blue), AL2 (yellow), AL3 (green), AL4 (red). (TIF) [file pone.0166176.s001.TIF]

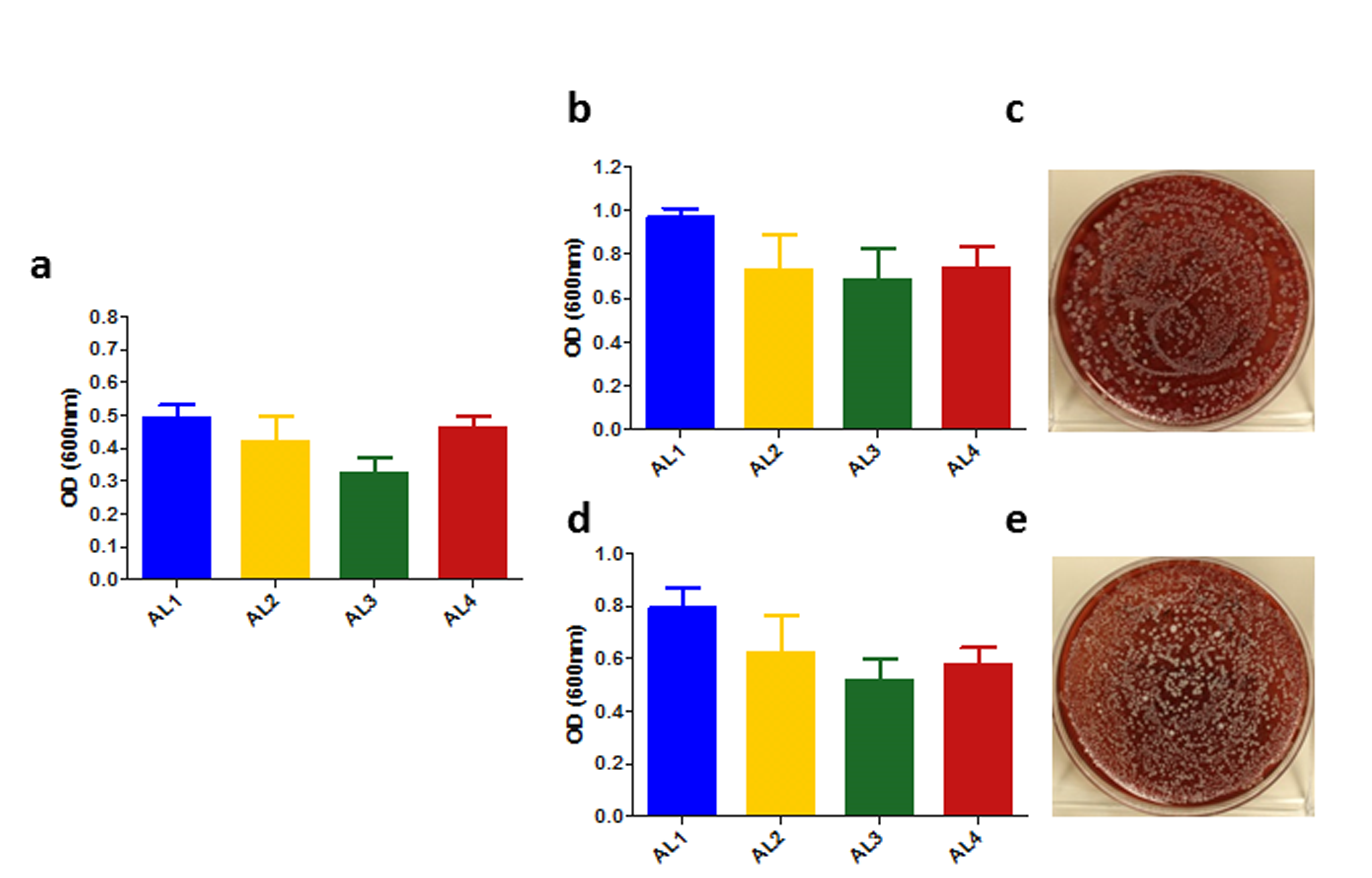

Supplement: S2 Fig — Bacterial culture and BTM preparation (a) Bacterial growth following 24 hours of incubation in MHBII broth (n = 7/AL). Bacterial pellets were then split and resuspended in either a-MEM or KM and incubated for an additional 24 hours to make BTM. (n = 7/AL/media) (b) MEM-BTM showed no significant differences between anatomical locations in bacterial density following incubation. (c) A representative image of colonies following incubation in MEM-BTM that retained a poly-microbial population. (d) KM-BTM also exhibited no significant differences in bacterial density between anatomical location and a diverse population following incubation in KM. (e) A representative image of colonies following incubation in MEM-BTM that retained a poly-microbial population. Images of 1–10,000 dilutions are shown. (TIF) [file pone.0166176.s002.TIF]
