# Supplementary material for: Initial Characterization of the Pig Skin Bacteriome and Its Effect on In Vitro Models of Wound Healing
Source: PLoS One. 2016 Nov 8;11(11):e0166176. doi: 10.1371/journal.pone.0166176 (PMC5100914; doi:10.1371/journal.pone.0166176)
Supplement: S1 Table — Each of the 518 identified species in relative abundance from each anatomical location and the average is shown to the lowest identified classification. (DOCX) [file pone.0166176.s003.docx]

|  | % Of species | | | | |
| --- | --- | --- | --- | --- | --- |
| Species Identified | Average | AL1 | AL2 | AL3 | AL4 |
| Unassigned;Other;Other;Other;Other;Other;Other | 1.8109 | 1.5386 | 1.7871 | 1.8527 | 2.0652 |
| k__Archaea;p__Euryarchaeota;c__Methanobacteria;o__Methanobacteriales;f__Methanobacteriaceae;g__Methanobrevibacter;s__ | 0.0252 | 0.0452 | 0.0271 | 0.0077 | 0.0209 |
| k__Archaea;p__Euryarchaeota;c__Methanobacteria;o__Methanobacteriales;f__Methanobacteriaceae;g__Methanosphaera;s__ | 0.0011 | 0.0032 | 0.0000 | 0.0000 | 0.0011 |
| k__Archaea;p__Euryarchaeota;c__Thermoplasmata;o__E2;f__[Methanomassiliicoccaceae];g__vadinCA11;s__ | 0.0084 | 0.0061 | 0.0063 | 0.0063 | 0.0150 |
| k__Bacteria;p__Acidobacteria;c__Acidobacteria-6;o__iii1-15;f__;g__;s__ | 0.0004 | 0.0000 | 0.0017 | 0.0000 | 0.0000 |
| k__Bacteria;p__Acidobacteria;c__[Chloracidobacteria];o__RB41;f__Ellin6075;g__;s__ | 0.0047 | 0.0038 | 0.0117 | 0.0021 | 0.0014 |
| k__Bacteria;p__Actinobacteria;c__Acidimicrobiia;o__Acidimicrobiales;f__;g__;s__ | 0.0046 | 0.0183 | 0.0000 | 0.0000 | 0.0000 |
| k__Bacteria;p__Actinobacteria;c__Acidimicrobiia;o__Acidimicrobiales;f__C111;g__;s__ | 0.0008 | 0.0000 | 0.0032 | 0.0000 | 0.0000 |
| k__Bacteria;p__Actinobacteria;c__Acidimicrobiia;o__Acidimicrobiales;f__Microthrixaceae;g__;s__ | 0.0009 | 0.0000 | 0.0000 | 0.0036 | 0.0000 |
| k__Bacteria;p__Actinobacteria;c__Actinobacteria;o__Actinomycetales;Other;Other;Other | 0.0594 | 0.0076 | 0.1609 | 0.0403 | 0.0290 |
| k__Bacteria;p__Actinobacteria;c__Actinobacteria;o__Actinomycetales;f__;g__;s__ | 0.0581 | 0.0000 | 0.1602 | 0.0528 | 0.0193 |
| k__Bacteria;p__Actinobacteria;c__Actinobacteria;o__Actinomycetales;f__Actinomycetaceae;g__;s__ | 0.0046 | 0.0000 | 0.0123 | 0.0014 | 0.0047 |
| k__Bacteria;p__Actinobacteria;c__Actinobacteria;o__Actinomycetales;f__Actinomycetaceae;g__Actinobaculum;s__ | 0.0002 | 0.0000 | 0.0000 | 0.0000 | 0.0009 |
| k__Bacteria;p__Actinobacteria;c__Actinobacteria;o__Actinomycetales;f__Actinomycetaceae;g__Actinomyces;s__ | 0.0396 | 0.1126 | 0.0265 | 0.0146 | 0.0046 |
| k__Bacteria;p__Actinobacteria;c__Actinobacteria;o__Actinomycetales;f__Actinomycetaceae;g__Trueperella;s__ | 0.0179 | 0.0310 | 0.0362 | 0.0045 | 0.0000 |
| k__Bacteria;p__Actinobacteria;c__Actinobacteria;o__Actinomycetales;f__Beutenbergiaceae;g__Salana;s__multivorans | 0.0029 | 0.0000 | 0.0017 | 0.0099 | 0.0000 |
| k__Bacteria;p__Actinobacteria;c__Actinobacteria;o__Actinomycetales;f__Bogoriellaceae;g__Georgenia;s__ | 0.0425 | 0.0051 | 0.0952 | 0.0607 | 0.0090 |
| k__Bacteria;p__Actinobacteria;c__Actinobacteria;o__Actinomycetales;f__Brevibacteriaceae;g__Brevibacterium;s__ | 0.1923 | 0.0330 | 0.3926 | 0.3250 | 0.0185 |
| k__Bacteria;p__Actinobacteria;c__Actinobacteria;o__Actinomycetales;f__Cellulomonadaceae;Other;Other | 0.0001 | 0.0000 | 0.0000 | 0.0000 | 0.0006 |
| k__Bacteria;p__Actinobacteria;c__Actinobacteria;o__Actinomycetales;f__Cellulomonadaceae;g__Cellulomonas;s__ | 0.0004 | 0.0003 | 0.0002 | 0.0003 | 0.0006 |
| k__Bacteria;p__Actinobacteria;c__Actinobacteria;o__Actinomycetales;f__Corynebacteriaceae;g__Corynebacterium;s__ | 0.3196 | 0.2915 | 0.5852 | 0.2921 | 0.1096 |
| k__Bacteria;p__Actinobacteria;c__Actinobacteria;o__Actinomycetales;f__Corynebacteriaceae;g__Corynebacterium;s__durum | 0.0001 | 0.0000 | 0.0000 | 0.0005 | 0.0000 |
| k__Bacteria;p__Actinobacteria;c__Actinobacteria;o__Actinomycetales;f__Corynebacteriaceae;g__Corynebacterium;s__variabile | 0.0093 | 0.0193 | 0.0114 | 0.0010 | 0.0055 |
| k__Bacteria;p__Actinobacteria;c__Actinobacteria;o__Actinomycetales;f__Dermabacteraceae;Other;Other | 0.0056 | 0.0014 | 0.0081 | 0.0126 | 0.0002 |
| k__Bacteria;p__Actinobacteria;c__Actinobacteria;o__Actinomycetales;f__Dermabacteraceae;g__;s__ | 0.0011 | 0.0008 | 0.0017 | 0.0018 | 0.0000 |
| k__Bacteria;p__Actinobacteria;c__Actinobacteria;o__Actinomycetales;f__Dermabacteraceae;g__Brachybacterium;Other | 0.0001 | 0.0000 | 0.0000 | 0.0002 | 0.0000 |
| k__Bacteria;p__Actinobacteria;c__Actinobacteria;o__Actinomycetales;f__Dermabacteraceae;g__Brachybacterium;s__ | 0.9974 | 0.3090 | 1.6795 | 1.8848 | 0.1163 |
| k__Bacteria;p__Actinobacteria;c__Actinobacteria;o__Actinomycetales;f__Dermabacteraceae;g__Brachybacterium;s__conglomeratum | 0.0001 | 0.0000 | 0.0005 | 0.0000 | 0.0000 |
| k__Bacteria;p__Actinobacteria;c__Actinobacteria;o__Actinomycetales;f__Dermacoccaceae;g__Dermacoccus;s__ | 0.1102 | 0.0418 | 0.2014 | 0.1959 | 0.0018 |
| k__Bacteria;p__Actinobacteria;c__Actinobacteria;o__Actinomycetales;f__Dermatophilaceae;Other;Other | 0.0002 | 0.0000 | 0.0005 | 0.0003 | 0.0000 |
| k__Bacteria;p__Actinobacteria;c__Actinobacteria;o__Actinomycetales;f__Dermatophilaceae;g__Dermatophilus;s__ | 0.0018 | 0.0036 | 0.0025 | 0.0003 | 0.0009 |
| k__Bacteria;p__Actinobacteria;c__Actinobacteria;o__Actinomycetales;f__Dietziaceae;g__Dietzia;s__ | 0.0671 | 0.0035 | 0.1524 | 0.1111 | 0.0015 |
| k__Bacteria;p__Actinobacteria;c__Actinobacteria;o__Actinomycetales;f__Frankiaceae;g__;s__ | 0.0003 | 0.0000 | 0.0012 | 0.0000 | 0.0000 |
| k__Bacteria;p__Actinobacteria;c__Actinobacteria;o__Actinomycetales;f__Geodermatophilaceae;Other;Other | 0.0002 | 0.0000 | 0.0000 | 0.0000 | 0.0007 |
| k__Bacteria;p__Actinobacteria;c__Actinobacteria;o__Actinomycetales;f__Intrasporangiaceae;Other;Other | 0.0117 | 0.0006 | 0.0274 | 0.0183 | 0.0006 |
| k__Bacteria;p__Actinobacteria;c__Actinobacteria;o__Actinomycetales;f__Intrasporangiaceae;g__;s__ | 0.0166 | 0.0243 | 0.0275 | 0.0124 | 0.0022 |
| k__Bacteria;p__Actinobacteria;c__Actinobacteria;o__Actinomycetales;f__Intrasporangiaceae;g__Phycicoccus;s__ | 0.0001 | 0.0000 | 0.0002 | 0.0000 | 0.0000 |
| k__Bacteria;p__Actinobacteria;c__Actinobacteria;o__Actinomycetales;f__Kineosporiaceae;g__;s__ | 0.0003 | 0.0000 | 0.0000 | 0.0012 | 0.0000 |
| k__Bacteria;p__Actinobacteria;c__Actinobacteria;o__Actinomycetales;f__Microbacteriaceae;Other;Other | 0.0004 | 0.0000 | 0.0005 | 0.0012 | 0.0000 |
| k__Bacteria;p__Actinobacteria;c__Actinobacteria;o__Actinomycetales;f__Microbacteriaceae;g__;s__ | 0.0005 | 0.0000 | 0.0007 | 0.0012 | 0.0000 |
| k__Bacteria;p__Actinobacteria;c__Actinobacteria;o__Actinomycetales;f__Microbacteriaceae;g__Leucobacter;s__ | 0.0164 | 0.0240 | 0.0197 | 0.0145 | 0.0076 |
| k__Bacteria;p__Actinobacteria;c__Actinobacteria;o__Actinomycetales;f__Microbacteriaceae;g__Pseudoclavibacter;Other | 0.0158 | 0.0217 | 0.0099 | 0.0230 | 0.0085 |
| k__Bacteria;p__Actinobacteria;c__Actinobacteria;o__Actinomycetales;f__Micrococcaceae;Other;Other | 0.0295 | 0.0084 | 0.0500 | 0.0567 | 0.0028 |
| k__Bacteria;p__Actinobacteria;c__Actinobacteria;o__Actinomycetales;f__Micrococcaceae;g__;s__ | 0.5968 | 0.5811 | 0.8690 | 0.8550 | 0.0821 |
| k__Bacteria;p__Actinobacteria;c__Actinobacteria;o__Actinomycetales;f__Micrococcaceae;g__Arthrobacter;s__ | 0.0040 | 0.0091 | 0.0042 | 0.0000 | 0.0026 |
| k__Bacteria;p__Actinobacteria;c__Actinobacteria;o__Actinomycetales;f__Micrococcaceae;g__Kocuria;Other | 0.0252 | 0.0074 | 0.0417 | 0.0492 | 0.0025 |
| k__Bacteria;p__Actinobacteria;c__Actinobacteria;o__Actinomycetales;f__Micrococcaceae;g__Kocuria;s__ | 0.0094 | 0.0020 | 0.0195 | 0.0125 | 0.0037 |
| k__Bacteria;p__Actinobacteria;c__Actinobacteria;o__Actinomycetales;f__Micrococcaceae;g__Kocuria;s__palustris | 0.0028 | 0.0008 | 0.0033 | 0.0062 | 0.0009 |
| k__Bacteria;p__Actinobacteria;c__Actinobacteria;o__Actinomycetales;f__Micrococcaceae;g__Kocuria;s__rhizophila | 6.9903 | 2.3561 | 12.7247 | 10.8729 | 2.0076 |
| k__Bacteria;p__Actinobacteria;c__Actinobacteria;o__Actinomycetales;f__Micrococcaceae;g__Microbispora;s__rosea | 0.0002 | 0.0000 | 0.0005 | 0.0003 | 0.0000 |
| k__Bacteria;p__Actinobacteria;c__Actinobacteria;o__Actinomycetales;f__Micrococcaceae;g__Micrococcus;Other | 0.0044 | 0.0022 | 0.0082 | 0.0069 | 0.0004 |
| k__Bacteria;p__Actinobacteria;c__Actinobacteria;o__Actinomycetales;f__Micrococcaceae;g__Micrococcus;s__ | 2.5625 | 0.7977 | 4.1422 | 5.1054 | 0.2046 |
| k__Bacteria;p__Actinobacteria;c__Actinobacteria;o__Actinomycetales;f__Micrococcaceae;g__Micrococcus;s__luteus | 0.0019 | 0.0000 | 0.0042 | 0.0035 | 0.0000 |
| k__Bacteria;p__Actinobacteria;c__Actinobacteria;o__Actinomycetales;f__Micrococcaceae;g__Renibacterium;s__ | 0.0001 | 0.0000 | 0.0000 | 0.0005 | 0.0000 |
| k__Bacteria;p__Actinobacteria;c__Actinobacteria;o__Actinomycetales;f__Micrococcaceae;g__Rothia;Other | 0.0007 | 0.0000 | 0.0012 | 0.0014 | 0.0002 |
| k__Bacteria;p__Actinobacteria;c__Actinobacteria;o__Actinomycetales;f__Micrococcaceae;g__Rothia;s__mucilaginosa | 0.0030 | 0.0000 | 0.0110 | 0.0012 | 0.0000 |
| k__Bacteria;p__Actinobacteria;c__Actinobacteria;o__Actinomycetales;f__Micrococcaceae;g__Rothia;s__nasimurium | 0.0001 | 0.0000 | 0.0002 | 0.0000 | 0.0000 |
| k__Bacteria;p__Actinobacteria;c__Actinobacteria;o__Actinomycetales;f__Micromonosporaceae;g__;s__ | 0.0001 | 0.0000 | 0.0000 | 0.0003 | 0.0000 |
| k__Bacteria;p__Actinobacteria;c__Actinobacteria;o__Actinomycetales;f__Mycobacteriaceae;g__Mycobacterium;s__ | 0.0007 | 0.0014 | 0.0015 | 0.0000 | 0.0000 |
| k__Bacteria;p__Actinobacteria;c__Actinobacteria;o__Actinomycetales;f__Nocardiaceae;Other;Other | 0.0001 | 0.0000 | 0.0002 | 0.0000 | 0.0000 |
| k__Bacteria;p__Actinobacteria;c__Actinobacteria;o__Actinomycetales;f__Nocardiaceae;g__Rhodococcus;s__fascians | 0.0010 | 0.0000 | 0.0002 | 0.0036 | 0.0000 |
| k__Bacteria;p__Actinobacteria;c__Actinobacteria;o__Actinomycetales;f__Nocardioidaceae;g__;s__ | 0.0015 | 0.0032 | 0.0000 | 0.0000 | 0.0027 |
| k__Bacteria;p__Actinobacteria;c__Actinobacteria;o__Actinomycetales;f__Nocardioidaceae;g__Aeromicrobium;s__ | 0.0005 | 0.0005 | 0.0000 | 0.0000 | 0.0015 |
| k__Bacteria;p__Actinobacteria;c__Actinobacteria;o__Actinomycetales;f__Nocardioidaceae;g__Nocardioides;s__ | 0.0001 | 0.0000 | 0.0000 | 0.0000 | 0.0003 |
| k__Bacteria;p__Actinobacteria;c__Actinobacteria;o__Actinomycetales;f__Nocardioidaceae;g__Propionicimonas;s__ | 0.0001 | 0.0000 | 0.0002 | 0.0000 | 0.0000 |
| k__Bacteria;p__Actinobacteria;c__Actinobacteria;o__Actinomycetales;f__Promicromonosporaceae;Other;Other | 0.0001 | 0.0000 | 0.0002 | 0.0000 | 0.0000 |
| k__Bacteria;p__Actinobacteria;c__Actinobacteria;o__Actinomycetales;f__Propionibacteriaceae;g__Propionibacterium;s__acnes | 0.0045 | 0.0037 | 0.0107 | 0.0007 | 0.0027 |
| k__Bacteria;p__Actinobacteria;c__Actinobacteria;o__Actinomycetales;f__Propionibacteriaceae;g__Tessaracoccus;s__ | 0.0006 | 0.0000 | 0.0025 | 0.0000 | 0.0000 |
| k__Bacteria;p__Actinobacteria;c__Actinobacteria;o__Actinomycetales;f__Pseudonocardiaceae;g__Amycolatopsis;s__ | 0.0002 | 0.0000 | 0.0006 | 0.0000 | 0.0000 |
| k__Bacteria;p__Actinobacteria;c__Actinobacteria;o__Actinomycetales;f__Pseudonocardiaceae;g__Saccharopolyspora;s__ | 0.0002 | 0.0000 | 0.0006 | 0.0000 | 0.0000 |
| k__Bacteria;p__Actinobacteria;c__Actinobacteria;o__Actinomycetales;f__Pseudonocardiaceae;g__Saccharopolyspora;s__hirsuta | 0.0002 | 0.0000 | 0.0000 | 0.0000 | 0.0008 |
| k__Bacteria;p__Actinobacteria;c__Actinobacteria;o__Actinomycetales;f__Ruaniaceae;Other;Other | 0.0021 | 0.0000 | 0.0080 | 0.0003 | 0.0000 |
| k__Bacteria;p__Actinobacteria;c__Actinobacteria;o__Actinomycetales;f__Sanguibacteraceae;g__Sanguibacter;s__ | 0.0017 | 0.0000 | 0.0002 | 0.0000 | 0.0067 |
| k__Bacteria;p__Actinobacteria;c__Actinobacteria;o__Actinomycetales;f__Sporichthyaceae;g__;s__ | 0.0003 | 0.0000 | 0.0000 | 0.0012 | 0.0000 |
| k__Bacteria;p__Actinobacteria;c__Actinobacteria;o__Actinomycetales;f__Streptomycetaceae;Other;Other | 0.0412 | 0.1563 | 0.0070 | 0.0009 | 0.0006 |
| k__Bacteria;p__Actinobacteria;c__Actinobacteria;o__Actinomycetales;f__Streptomycetaceae;g__Streptomyces;Other | 0.0001 | 0.0000 | 0.0002 | 0.0002 | 0.0000 |
| k__Bacteria;p__Actinobacteria;c__Actinobacteria;o__Actinomycetales;f__Streptomycetaceae;g__Streptomyces;s__ | 0.0001 | 0.0000 | 0.0000 | 0.0002 | 0.0000 |
| k__Bacteria;p__Actinobacteria;c__Actinobacteria;o__Actinomycetales;f__Yaniellaceae;g__Yaniella;s__ | 0.0001 | 0.0000 | 0.0000 | 0.0000 | 0.0003 |
| k__Bacteria;p__Actinobacteria;c__Actinobacteria;o__Bifidobacteriales;f__Bifidobacteriaceae;g__;s__ | 0.0058 | 0.0196 | 0.0016 | 0.0000 | 0.0021 |
| k__Bacteria;p__Actinobacteria;c__Actinobacteria;o__Bifidobacteriales;f__Bifidobacteriaceae;g__Bifidobacterium;Other | 0.0001 | 0.0003 | 0.0000 | 0.0000 | 0.0000 |
| k__Bacteria;p__Actinobacteria;c__Actinobacteria;o__Bifidobacteriales;f__Bifidobacteriaceae;g__Bifidobacterium;s__ | 0.0761 | 0.1424 | 0.0770 | 0.0571 | 0.0280 |
| k__Bacteria;p__Actinobacteria;c__Actinobacteria;o__Bifidobacteriales;f__Bifidobacteriaceae;g__Bifidobacterium;s__pseudolongum | 0.0705 | 0.0463 | 0.0775 | 0.0898 | 0.0685 |
| k__Bacteria;p__Actinobacteria;c__Actinobacteria;o__Bifidobacteriales;f__Bifidobacteriaceae;g__Scardovia;s__ | 0.0019 | 0.0037 | 0.0040 | 0.0000 | 0.0000 |
| k__Bacteria;p__Actinobacteria;c__Coriobacteriia;o__Coriobacteriales;f__Coriobacteriaceae;g__;s__ | 0.5074 | 0.6657 | 0.4791 | 0.3663 | 0.5184 |
| k__Bacteria;p__Actinobacteria;c__Coriobacteriia;o__Coriobacteriales;f__Coriobacteriaceae;g__Adlercreutzia;s__ | 0.0187 | 0.0095 | 0.0248 | 0.0223 | 0.0183 |
| k__Bacteria;p__Actinobacteria;c__Coriobacteriia;o__Coriobacteriales;f__Coriobacteriaceae;g__Atopobium;s__ | 0.0279 | 0.0878 | 0.0181 | 0.0056 | 0.0000 |
| k__Bacteria;p__Actinobacteria;c__Coriobacteriia;o__Coriobacteriales;f__Coriobacteriaceae;g__Collinsella;s__aerofaciens | 0.2027 | 0.3561 | 0.1791 | 0.1449 | 0.1307 |
| k__Bacteria;p__Actinobacteria;c__Coriobacteriia;o__Coriobacteriales;f__Coriobacteriaceae;g__Collinsella;s__stercoris | 0.0698 | 0.1172 | 0.0498 | 0.0772 | 0.0348 |
| k__Bacteria;p__Actinobacteria;c__Coriobacteriia;o__Coriobacteriales;f__Coriobacteriaceae;g__Slackia;s__ | 0.0066 | 0.0053 | 0.0112 | 0.0085 | 0.0015 |
| k__Bacteria;p__Actinobacteria;c__OPB41;o__;f__;g__;s__ | 0.0002 | 0.0000 | 0.0000 | 0.0007 | 0.0000 |
| k__Bacteria;p__Actinobacteria;c__Thermoleophilia;o__Gaiellales;f__;g__;s__ | 0.0008 | 0.0033 | 0.0000 | 0.0000 | 0.0000 |
| k__Bacteria;p__Actinobacteria;c__Thermoleophilia;o__Gaiellales;f__Gaiellaceae;g__;s__ | 0.0006 | 0.0026 | 0.0000 | 0.0000 | 0.0000 |
| k__Bacteria;p__Actinobacteria;c__Thermoleophilia;o__Solirubrobacterales;f__;g__;s__ | 0.0013 | 0.0000 | 0.0053 | 0.0000 | 0.0000 |
| k__Bacteria;p__Actinobacteria;c__Thermoleophilia;o__Solirubrobacterales;f__Solirubrobacteraceae;g__;s__ | 0.0002 | 0.0000 | 0.0000 | 0.0000 | 0.0009 |
| k__Bacteria;p__Bacteroidetes;c__Bacteroidia;o__Bacteroidales;Other;Other;Other | 0.0014 | 0.0011 | 0.0002 | 0.0023 | 0.0020 |
| k__Bacteria;p__Bacteroidetes;c__Bacteroidia;o__Bacteroidales;f__;g__;s__ | 3.2721 | 2.9711 | 1.8756 | 3.7356 | 4.5062 |
| k__Bacteria;p__Bacteroidetes;c__Bacteroidia;o__Bacteroidales;f__BS11;g__;s__ | 0.0013 | 0.0039 | 0.0012 | 0.0000 | 0.0000 |
| k__Bacteria;p__Bacteroidetes;c__Bacteroidia;o__Bacteroidales;f__Bacteroidaceae;g__Bacteroides;Other | 0.0001 | 0.0003 | 0.0000 | 0.0002 | 0.0000 |
| k__Bacteria;p__Bacteroidetes;c__Bacteroidia;o__Bacteroidales;f__Bacteroidaceae;g__Bacteroides;s__ | 0.5997 | 0.8307 | 0.4052 | 0.4547 | 0.7083 |
| k__Bacteria;p__Bacteroidetes;c__Bacteroidia;o__Bacteroidales;f__Bacteroidaceae;g__Bacteroides;s__barnesiae | 0.0003 | 0.0002 | 0.0000 | 0.0002 | 0.0009 |
| k__Bacteria;p__Bacteroidetes;c__Bacteroidia;o__Bacteroidales;f__Bacteroidaceae;g__Bacteroides;s__coprophilus | 0.0036 | 0.0095 | 0.0000 | 0.0021 | 0.0029 |
| k__Bacteria;p__Bacteroidetes;c__Bacteroidia;o__Bacteroidales;f__Bacteroidaceae;g__Bacteroides;s__fragilis | 0.0060 | 0.0053 | 0.0059 | 0.0052 | 0.0077 |
| k__Bacteria;p__Bacteroidetes;c__Bacteroidia;o__Bacteroidales;f__Bacteroidaceae;g__Bacteroides;s__ovatus | 0.0001 | 0.0000 | 0.0000 | 0.0000 | 0.0005 |
| k__Bacteria;p__Bacteroidetes;c__Bacteroidia;o__Bacteroidales;f__Bacteroidaceae;g__Bacteroides;s__plebeius | 0.0001 | 0.0000 | 0.0000 | 0.0000 | 0.0002 |
| k__Bacteria;p__Bacteroidetes;c__Bacteroidia;o__Bacteroidales;f__Bacteroidaceae;g__Bacteroides;s__uniformis | 0.0002 | 0.0002 | 0.0000 | 0.0002 | 0.0003 |
| k__Bacteria;p__Bacteroidetes;c__Bacteroidia;o__Bacteroidales;f__Porphyromonadaceae;Other;Other | 0.0002 | 0.0002 | 0.0000 | 0.0002 | 0.0002 |
| k__Bacteria;p__Bacteroidetes;c__Bacteroidia;o__Bacteroidales;f__Porphyromonadaceae;g__;s__ | 0.0008 | 0.0000 | 0.0000 | 0.0000 | 0.0032 |
| k__Bacteria;p__Bacteroidetes;c__Bacteroidia;o__Bacteroidales;f__Porphyromonadaceae;g__Dysgonomonas;s__ | 0.0002 | 0.0000 | 0.0010 | 0.0000 | 0.0000 |
| k__Bacteria;p__Bacteroidetes;c__Bacteroidia;o__Bacteroidales;f__Porphyromonadaceae;g__Paludibacter;s__ | 0.1228 | 0.0980 | 0.1097 | 0.1553 | 0.1282 |
| k__Bacteria;p__Bacteroidetes;c__Bacteroidia;o__Bacteroidales;f__Porphyromonadaceae;g__Parabacteroides;s__ | 0.2551 | 0.2613 | 0.2002 | 0.2126 | 0.3461 |
| k__Bacteria;p__Bacteroidetes;c__Bacteroidia;o__Bacteroidales;f__Porphyromonadaceae;g__Parabacteroides;s__distasonis | 0.0007 | 0.0027 | 0.0000 | 0.0000 | 0.0000 |
| k__Bacteria;p__Bacteroidetes;c__Bacteroidia;o__Bacteroidales;f__Porphyromonadaceae;g__Porphyromonas;s__ | 0.0919 | 0.2309 | 0.0850 | 0.0456 | 0.0062 |
| k__Bacteria;p__Bacteroidetes;c__Bacteroidia;o__Bacteroidales;f__Porphyromonadaceae;g__Porphyromonas;s__endodontalis | 0.0212 | 0.0454 | 0.0325 | 0.0018 | 0.0052 |
| k__Bacteria;p__Bacteroidetes;c__Bacteroidia;o__Bacteroidales;f__Porphyromonadaceae;g__Tannerella;s__ | 0.0009 | 0.0000 | 0.0013 | 0.0021 | 0.0000 |
| k__Bacteria;p__Bacteroidetes;c__Bacteroidia;o__Bacteroidales;f__Prevotellaceae;Other;Other | 0.0002 | 0.0000 | 0.0006 | 0.0000 | 0.0000 |
| k__Bacteria;p__Bacteroidetes;c__Bacteroidia;o__Bacteroidales;f__Prevotellaceae;g__;s__ | 0.0017 | 0.0012 | 0.0031 | 0.0009 | 0.0017 |
| k__Bacteria;p__Bacteroidetes;c__Bacteroidia;o__Bacteroidales;f__Prevotellaceae;g__Prevotella;Other | 0.0007 | 0.0015 | 0.0002 | 0.0002 | 0.0010 |
| k__Bacteria;p__Bacteroidetes;c__Bacteroidia;o__Bacteroidales;f__Prevotellaceae;g__Prevotella;s__ | 7.1522 | 7.7280 | 5.7014 | 6.4009 | 8.7784 |
| k__Bacteria;p__Bacteroidetes;c__Bacteroidia;o__Bacteroidales;f__Prevotellaceae;g__Prevotella;s__copri | 2.3576 | 2.6545 | 1.2644 | 2.5319 | 2.9797 |
| k__Bacteria;p__Bacteroidetes;c__Bacteroidia;o__Bacteroidales;f__Prevotellaceae;g__Prevotella;s__melaninogenica | 0.0122 | 0.0143 | 0.0101 | 0.0088 | 0.0156 |
| k__Bacteria;p__Bacteroidetes;c__Bacteroidia;o__Bacteroidales;f__Prevotellaceae;g__Prevotella;s__nanceiensis | 0.0006 | 0.0002 | 0.0005 | 0.0004 | 0.0013 |
| k__Bacteria;p__Bacteroidetes;c__Bacteroidia;o__Bacteroidales;f__Prevotellaceae;g__Prevotella;s__nigrescens | 0.0039 | 0.0155 | 0.0000 | 0.0000 | 0.0000 |
| k__Bacteria;p__Bacteroidetes;c__Bacteroidia;o__Bacteroidales;f__Prevotellaceae;g__Prevotella;s__pallens | 0.0001 | 0.0000 | 0.0000 | 0.0000 | 0.0002 |
| k__Bacteria;p__Bacteroidetes;c__Bacteroidia;o__Bacteroidales;f__Prevotellaceae;g__Prevotella;s__stercorea | 0.2107 | 0.2107 | 0.0527 | 0.3039 | 0.2754 |
| k__Bacteria;p__Bacteroidetes;c__Bacteroidia;o__Bacteroidales;f__RF16;g__;s__ | 0.7765 | 0.7359 | 0.5058 | 0.7922 | 1.0722 |
| k__Bacteria;p__Bacteroidetes;c__Bacteroidia;o__Bacteroidales;f__Rikenellaceae;Other;Other | 0.0001 | 0.0000 | 0.0005 | 0.0000 | 0.0000 |
| k__Bacteria;p__Bacteroidetes;c__Bacteroidia;o__Bacteroidales;f__Rikenellaceae;g__;s__ | 0.0431 | 0.0515 | 0.0478 | 0.0086 | 0.0645 |
| k__Bacteria;p__Bacteroidetes;c__Bacteroidia;o__Bacteroidales;f__S24-7;g__;s__ | 2.0178 | 1.8769 | 1.6118 | 1.9037 | 2.6789 |
| k__Bacteria;p__Bacteroidetes;c__Bacteroidia;o__Bacteroidales;f__[Odoribacteraceae];g__Butyricimonas;s__ | 0.0002 | 0.0000 | 0.0000 | 0.0007 | 0.0000 |
| k__Bacteria;p__Bacteroidetes;c__Bacteroidia;o__Bacteroidales;f__[Paraprevotellaceae];Other;Other | 0.0003 | 0.0000 | 0.0000 | 0.0014 | 0.0000 |
| k__Bacteria;p__Bacteroidetes;c__Bacteroidia;o__Bacteroidales;f__[Paraprevotellaceae];g__;s__ | 1.2657 | 0.9844 | 0.5623 | 1.9738 | 1.5424 |
| k__Bacteria;p__Bacteroidetes;c__Bacteroidia;o__Bacteroidales;f__[Paraprevotellaceae];g__CF231;s__ | 0.3128 | 0.3991 | 0.1905 | 0.2613 | 0.4004 |
| k__Bacteria;p__Bacteroidetes;c__Bacteroidia;o__Bacteroidales;f__[Paraprevotellaceae];g__YRC22;s__ | 0.2023 | 0.1903 | 0.1979 | 0.2754 | 0.1456 |
| k__Bacteria;p__Bacteroidetes;c__Bacteroidia;o__Bacteroidales;f__[Paraprevotellaceae];g__[Prevotella];s__ | 1.6889 | 1.4889 | 1.5053 | 1.7479 | 2.0135 |
| k__Bacteria;p__Bacteroidetes;c__Bacteroidia;o__Bacteroidales;f__[Paraprevotellaceae];g__[Prevotella];s__tannerae | 0.0001 | 0.0000 | 0.0002 | 0.0000 | 0.0000 |
| k__Bacteria;p__Bacteroidetes;c__Bacteroidia;o__Bacteroidales;f__p-2534-18B5;g__;s__ | 0.0036 | 0.0074 | 0.0055 | 0.0000 | 0.0014 |
| k__Bacteria;p__Bacteroidetes;c__Cytophagia;o__Cytophagales;f__Cytophagaceae;g__;s__ | 0.0011 | 0.0000 | 0.0044 | 0.0000 | 0.0000 |
| k__Bacteria;p__Bacteroidetes;c__Cytophagia;o__Cytophagales;f__Cytophagaceae;g__Dyadobacter;s__ | 0.0025 | 0.0068 | 0.0010 | 0.0021 | 0.0000 |
| k__Bacteria;p__Bacteroidetes;c__Cytophagia;o__Cytophagales;f__Cytophagaceae;g__Hymenobacter;s__ | 0.0003 | 0.0012 | 0.0000 | 0.0000 | 0.0000 |
| k__Bacteria;p__Bacteroidetes;c__Cytophagia;o__Cytophagales;f__Cytophagaceae;g__Leadbetterella;s__ | 0.0003 | 0.0000 | 0.0000 | 0.0012 | 0.0000 |
| k__Bacteria;p__Bacteroidetes;c__Flavobacteriia;o__Flavobacteriales;f__Flavobacteriaceae;g__;s__ | 0.0620 | 0.0083 | 0.0701 | 0.1451 | 0.0246 |
| k__Bacteria;p__Bacteroidetes;c__Flavobacteriia;o__Flavobacteriales;f__Flavobacteriaceae;g__Capnocytophaga;s__ | 0.0045 | 0.0082 | 0.0012 | 0.0083 | 0.0003 |
| k__Bacteria;p__Bacteroidetes;c__Flavobacteriia;o__Flavobacteriales;f__Flavobacteriaceae;g__Flavobacterium;s__ | 0.0007 | 0.0014 | 0.0012 | 0.0000 | 0.0000 |
| k__Bacteria;p__Bacteroidetes;c__Flavobacteriia;o__Flavobacteriales;f__[Weeksellaceae];g__;s__ | 0.0622 | 0.0384 | 0.0820 | 0.0926 | 0.0358 |
| k__Bacteria;p__Bacteroidetes;c__Flavobacteriia;o__Flavobacteriales;f__[Weeksellaceae];g__Chryseobacterium;s__ | 0.1122 | 0.0671 | 0.1559 | 0.2187 | 0.0071 |
| k__Bacteria;p__Bacteroidetes;c__Flavobacteriia;o__Flavobacteriales;f__[Weeksellaceae];g__Cloacibacterium;s__ | 0.0002 | 0.0010 | 0.0000 | 0.0000 | 0.0000 |
| k__Bacteria;p__Bacteroidetes;c__Flavobacteriia;o__Flavobacteriales;f__[Weeksellaceae];g__Wautersiella;s__ | 0.0022 | 0.0035 | 0.0054 | 0.0000 | 0.0000 |
| k__Bacteria;p__Bacteroidetes;c__Sphingobacteriia;o__Sphingobacteriales;f__Sphingobacteriaceae;g__;s__ | 0.0058 | 0.0135 | 0.0050 | 0.0048 | 0.0000 |
| k__Bacteria;p__Bacteroidetes;c__Sphingobacteriia;o__Sphingobacteriales;f__Sphingobacteriaceae;g__Sphingobacterium;s__ | 0.0649 | 0.0117 | 0.1033 | 0.1388 | 0.0060 |
| k__Bacteria;p__Bacteroidetes;c__Sphingobacteriia;o__Sphingobacteriales;f__Sphingobacteriaceae;g__Sphingobacterium;s__faecium | 0.0019 | 0.0000 | 0.0022 | 0.0055 | 0.0000 |
| k__Bacteria;p__Bacteroidetes;c__Sphingobacteriia;o__Sphingobacteriales;f__Sphingobacteriaceae;g__Sphingobacterium;s__mizutaii | 0.0027 | 0.0002 | 0.0052 | 0.0048 | 0.0005 |
| k__Bacteria;p__Bacteroidetes;c__[Saprospirae];o__[Saprospirales];f__Chitinophagaceae;g__;s__ | 0.0058 | 0.0060 | 0.0025 | 0.0103 | 0.0045 |
| k__Bacteria;p__Bacteroidetes;c__[Saprospirae];o__[Saprospirales];f__Chitinophagaceae;g__Niabella;s__ | 0.0004 | 0.0000 | 0.0017 | 0.0000 | 0.0000 |
| k__Bacteria;p__Bacteroidetes;c__[Saprospirae];o__[Saprospirales];f__Chitinophagaceae;g__Sediminibacterium;s__ | 0.0103 | 0.0117 | 0.0094 | 0.0109 | 0.0090 |
| k__Bacteria;p__Chlamydiae;c__Chlamydiia;o__Chlamydiales;f__Chlamydiaceae;g__Chlamydia;Other | 0.0286 | 0.0196 | 0.0319 | 0.0284 | 0.0347 |
| k__Bacteria;p__Chlamydiae;c__Chlamydiia;o__Chlamydiales;f__Chlamydiaceae;g__Chlamydia;s__ | 0.0001 | 0.0000 | 0.0002 | 0.0000 | 0.0000 |
| k__Bacteria;p__Chloroflexi;c__Chloroflexi;o__Herpetosiphonales;f__;g__;s__ | 0.0009 | 0.0000 | 0.0037 | 0.0000 | 0.0000 |
| k__Bacteria;p__Chloroflexi;c__Thermomicrobia;o__JG30-KF-CM45;f__;g__;s__ | 0.0154 | 0.0096 | 0.0277 | 0.0224 | 0.0020 |
| k__Bacteria;p__Cyanobacteria;c__4C0d-2;o__MLE1-12;f__;g__;s__ | 0.0190 | 0.0526 | 0.0113 | 0.0063 | 0.0056 |
| k__Bacteria;p__Cyanobacteria;c__4C0d-2;o__YS2;f__;g__;s__ | 1.2595 | 1.0029 | 0.6371 | 1.7665 | 1.6314 |
| k__Bacteria;p__Cyanobacteria;c__Chloroplast;o__Streptophyta;f__;g__;s__ | 0.0590 | 0.1625 | 0.0690 | 0.0012 | 0.0031 |
| k__Bacteria;p__Deferribacteres;c__Deferribacteres;o__Deferribacterales;f__Deferribacteraceae;g__Mucispirillum;s__schaedleri | 0.0135 | 0.0339 | 0.0036 | 0.0056 | 0.0111 |
| k__Bacteria;p__Elusimicrobia;c__Elusimicrobia;o__Elusimicrobiales;f__Elusimicrobiaceae;g__;s__ | 0.0185 | 0.0190 | 0.0126 | 0.0113 | 0.0311 |
| k__Bacteria;p__FBP;c__;o__;f__;g__;s__ | 0.0029 | 0.0087 | 0.0018 | 0.0012 | 0.0000 |
| k__Bacteria;p__Fibrobacteres;c__Fibrobacteria;o__Fibrobacterales;f__Fibrobacteraceae;g__Fibrobacter;s__ | 0.0274 | 0.0012 | 0.0107 | 0.0382 | 0.0595 |
| k__Bacteria;p__Fibrobacteres;c__Fibrobacteria;o__Fibrobacterales;f__Fibrobacteraceae;g__Fibrobacter;s__succinogenes | 0.0012 | 0.0000 | 0.0049 | 0.0000 | 0.0000 |
| k__Bacteria;p__Firmicutes;c__Bacilli;o__Bacillales;Other;Other;Other | 0.0001 | 0.0002 | 0.0000 | 0.0000 | 0.0000 |
| k__Bacteria;p__Firmicutes;c__Bacilli;o__Bacillales;f__;g__;s__ | 0.0013 | 0.0000 | 0.0000 | 0.0052 | 0.0000 |
| k__Bacteria;p__Firmicutes;c__Bacilli;o__Bacillales;f__Bacillaceae;Other;Other | 0.0011 | 0.0000 | 0.0042 | 0.0000 | 0.0000 |
| k__Bacteria;p__Firmicutes;c__Bacilli;o__Bacillales;f__Bacillaceae;g__Anoxybacillus;s__kestanbolensis | 0.0001 | 0.0000 | 0.0002 | 0.0000 | 0.0000 |
| k__Bacteria;p__Firmicutes;c__Bacilli;o__Bacillales;f__Bacillaceae;g__Bacillus;s__ | 0.0078 | 0.0114 | 0.0146 | 0.0052 | 0.0000 |
| k__Bacteria;p__Firmicutes;c__Bacilli;o__Bacillales;f__Bacillaceae;g__Bacillus;s__cereus | 0.0002 | 0.0000 | 0.0000 | 0.0000 | 0.0009 |
| k__Bacteria;p__Firmicutes;c__Bacilli;o__Bacillales;f__Bacillaceae;g__Geobacillus;s__ | 0.0003 | 0.0000 | 0.0012 | 0.0000 | 0.0000 |
| k__Bacteria;p__Firmicutes;c__Bacilli;o__Bacillales;f__Paenibacillaceae;g__Brevibacillus;Other | 0.0012 | 0.0000 | 0.0000 | 0.0002 | 0.0047 |
| k__Bacteria;p__Firmicutes;c__Bacilli;o__Bacillales;f__Paenibacillaceae;g__Cohnella;s__ | 0.0004 | 0.0000 | 0.0000 | 0.0000 | 0.0015 |
| k__Bacteria;p__Firmicutes;c__Bacilli;o__Bacillales;f__Planococcaceae;Other;Other | 0.0011 | 0.0016 | 0.0004 | 0.0025 | 0.0000 |
| k__Bacteria;p__Firmicutes;c__Bacilli;o__Bacillales;f__Planococcaceae;g__;s__ | 0.0053 | 0.0055 | 0.0146 | 0.0010 | 0.0003 |
| k__Bacteria;p__Firmicutes;c__Bacilli;o__Bacillales;f__Planococcaceae;g__Planomicrobium;s__ | 0.0006 | 0.0000 | 0.0018 | 0.0006 | 0.0000 |
| k__Bacteria;p__Firmicutes;c__Bacilli;o__Bacillales;f__Planococcaceae;g__Rummeliibacillus;s__ | 0.0190 | 0.0234 | 0.0429 | 0.0096 | 0.0000 |
| k__Bacteria;p__Firmicutes;c__Bacilli;o__Bacillales;f__Planococcaceae;g__Solibacillus;s__ | 0.0062 | 0.0027 | 0.0000 | 0.0220 | 0.0000 |
| k__Bacteria;p__Firmicutes;c__Bacilli;o__Bacillales;f__Staphylococcaceae;Other;Other | 0.0003 | 0.0000 | 0.0000 | 0.0000 | 0.0012 |
| k__Bacteria;p__Firmicutes;c__Bacilli;o__Bacillales;f__Staphylococcaceae;g__Jeotgalicoccus;s__ | 0.0006 | 0.0000 | 0.0000 | 0.0000 | 0.0023 |
| k__Bacteria;p__Firmicutes;c__Bacilli;o__Bacillales;f__Staphylococcaceae;g__Jeotgalicoccus;s__psychrophilus | 0.0087 | 0.0065 | 0.0284 | 0.0000 | 0.0000 |
| k__Bacteria;p__Firmicutes;c__Bacilli;o__Bacillales;f__Staphylococcaceae;g__Macrococcus;Other | 0.0001 | 0.0000 | 0.0000 | 0.0003 | 0.0000 |
| k__Bacteria;p__Firmicutes;c__Bacilli;o__Bacillales;f__Staphylococcaceae;g__Macrococcus;s__ | 0.0003 | 0.0003 | 0.0006 | 0.0002 | 0.0000 |
| k__Bacteria;p__Firmicutes;c__Bacilli;o__Bacillales;f__Staphylococcaceae;g__Macrococcus;s__caseolyticus | 0.1065 | 0.0831 | 0.1924 | 0.1206 | 0.0299 |
| k__Bacteria;p__Firmicutes;c__Bacilli;o__Bacillales;f__Staphylococcaceae;g__Staphylococcus;Other | 0.0007 | 0.0018 | 0.0000 | 0.0009 | 0.0000 |
| k__Bacteria;p__Firmicutes;c__Bacilli;o__Bacillales;f__Staphylococcaceae;g__Staphylococcus;s__ | 0.0291 | 0.0412 | 0.0370 | 0.0242 | 0.0140 |
| k__Bacteria;p__Firmicutes;c__Bacilli;o__Bacillales;f__Staphylococcaceae;g__Staphylococcus;s__aureus | 0.4947 | 0.9084 | 0.3764 | 0.4712 | 0.2226 |
| k__Bacteria;p__Firmicutes;c__Bacilli;o__Bacillales;f__Staphylococcaceae;g__Staphylococcus;s__epidermidis | 0.0171 | 0.0349 | 0.0134 | 0.0123 | 0.0080 |
| k__Bacteria;p__Firmicutes;c__Bacilli;o__Bacillales;f__Staphylococcaceae;g__Staphylococcus;s__equorum | 0.0001 | 0.0002 | 0.0002 | 0.0000 | 0.0000 |
| k__Bacteria;p__Firmicutes;c__Bacilli;o__Bacillales;f__Staphylococcaceae;g__Staphylococcus;s__sciuri | 0.0274 | 0.0251 | 0.0150 | 0.0355 | 0.0339 |
| k__Bacteria;p__Firmicutes;c__Bacilli;o__Bacillales;f__[Exiguobacteraceae];g__Exiguobacterium;s__ | 0.0563 | 0.1256 | 0.0661 | 0.0226 | 0.0110 |
| k__Bacteria;p__Firmicutes;c__Bacilli;o__Gemellales;f__Gemellaceae;g__;s__ | 0.0002 | 0.0008 | 0.0000 | 0.0000 | 0.0000 |
| k__Bacteria;p__Firmicutes;c__Bacilli;o__Gemellales;f__Gemellaceae;g__Gemella;s__ | 0.0224 | 0.0344 | 0.0153 | 0.0339 | 0.0058 |
| k__Bacteria;p__Firmicutes;c__Bacilli;o__Lactobacillales;Other;Other;Other | 0.0004 | 0.0005 | 0.0012 | 0.0000 | 0.0000 |
| k__Bacteria;p__Firmicutes;c__Bacilli;o__Lactobacillales;f__;g__;s__ | 0.0107 | 0.0411 | 0.0016 | 0.0000 | 0.0000 |
| k__Bacteria;p__Firmicutes;c__Bacilli;o__Lactobacillales;f__Aerococcaceae;g__;s__ | 0.0100 | 0.0278 | 0.0100 | 0.0000 | 0.0023 |
| k__Bacteria;p__Firmicutes;c__Bacilli;o__Lactobacillales;f__Aerococcaceae;g__Aerococcus;s__ | 0.0509 | 0.0603 | 0.0776 | 0.0471 | 0.0186 |
| k__Bacteria;p__Firmicutes;c__Bacilli;o__Lactobacillales;f__Aerococcaceae;g__Alloiococcus;s__ | 0.0012 | 0.0000 | 0.0037 | 0.0000 | 0.0012 |
| k__Bacteria;p__Firmicutes;c__Bacilli;o__Lactobacillales;f__Aerococcaceae;g__Alloiococcus;s__otitis | 0.0012 | 0.0000 | 0.0000 | 0.0050 | 0.0000 |
| k__Bacteria;p__Firmicutes;c__Bacilli;o__Lactobacillales;f__Aerococcaceae;g__Facklamia;s__ | 0.1411 | 0.3163 | 0.1223 | 0.0478 | 0.0781 |
| k__Bacteria;p__Firmicutes;c__Bacilli;o__Lactobacillales;f__Carnobacteriaceae;g__Desemzia;s__ | 0.0002 | 0.0000 | 0.0008 | 0.0000 | 0.0000 |
| k__Bacteria;p__Firmicutes;c__Bacilli;o__Lactobacillales;f__Carnobacteriaceae;g__Granulicatella;s__ | 0.0086 | 0.0137 | 0.0073 | 0.0024 | 0.0110 |
| k__Bacteria;p__Firmicutes;c__Bacilli;o__Lactobacillales;f__Carnobacteriaceae;g__Trichococcus;s__ | 0.0040 | 0.0000 | 0.0128 | 0.0012 | 0.0021 |
| k__Bacteria;p__Firmicutes;c__Bacilli;o__Lactobacillales;f__Enterococcaceae;Other;Other | 0.0009 | 0.0005 | 0.0006 | 0.0017 | 0.0007 |
| k__Bacteria;p__Firmicutes;c__Bacilli;o__Lactobacillales;f__Enterococcaceae;g__Enterococcus;s__ | 0.0119 | 0.0243 | 0.0142 | 0.0075 | 0.0018 |
| k__Bacteria;p__Firmicutes;c__Bacilli;o__Lactobacillales;f__Enterococcaceae;g__Vagococcus;s__ | 0.0136 | 0.0536 | 0.0007 | 0.0000 | 0.0000 |
| k__Bacteria;p__Firmicutes;c__Bacilli;o__Lactobacillales;f__Lactobacillaceae;Other;Other | 0.0010 | 0.0033 | 0.0005 | 0.0002 | 0.0000 |
| k__Bacteria;p__Firmicutes;c__Bacilli;o__Lactobacillales;f__Lactobacillaceae;g__;s__ | 0.0025 | 0.0102 | 0.0000 | 0.0000 | 0.0000 |
| k__Bacteria;p__Firmicutes;c__Bacilli;o__Lactobacillales;f__Lactobacillaceae;g__Lactobacillus;Other | 0.0025 | 0.0068 | 0.0027 | 0.0007 | 0.0000 |
| k__Bacteria;p__Firmicutes;c__Bacilli;o__Lactobacillales;f__Lactobacillaceae;g__Lactobacillus;s__ | 1.3867 | 2.9541 | 1.3504 | 0.6522 | 0.5903 |
| k__Bacteria;p__Firmicutes;c__Bacilli;o__Lactobacillales;f__Lactobacillaceae;g__Lactobacillus;s__agilis | 0.0003 | 0.0000 | 0.0012 | 0.0000 | 0.0000 |
| k__Bacteria;p__Firmicutes;c__Bacilli;o__Lactobacillales;f__Lactobacillaceae;g__Lactobacillus;s__brevis | 0.0006 | 0.0022 | 0.0000 | 0.0000 | 0.0000 |
| k__Bacteria;p__Firmicutes;c__Bacilli;o__Lactobacillales;f__Lactobacillaceae;g__Lactobacillus;s__mucosae | 0.0588 | 0.0595 | 0.0670 | 0.0469 | 0.0619 |
| k__Bacteria;p__Firmicutes;c__Bacilli;o__Lactobacillales;f__Lactobacillaceae;g__Lactobacillus;s__reuteri | 0.1888 | 0.1849 | 0.2732 | 0.1176 | 0.1794 |
| k__Bacteria;p__Firmicutes;c__Bacilli;o__Lactobacillales;f__Lactobacillaceae;g__Lactobacillus;s__ruminis | 0.0142 | 0.0380 | 0.0139 | 0.0015 | 0.0032 |
| k__Bacteria;p__Firmicutes;c__Bacilli;o__Lactobacillales;f__Lactobacillaceae;g__Lactobacillus;s__zeae | 0.0019 | 0.0073 | 0.0000 | 0.0000 | 0.0002 |
| k__Bacteria;p__Firmicutes;c__Bacilli;o__Lactobacillales;f__Lactobacillaceae;g__Pediococcus;s__acidilactici | 0.0006 | 0.0024 | 0.0000 | 0.0000 | 0.0000 |
| k__Bacteria;p__Firmicutes;c__Bacilli;o__Lactobacillales;f__Leuconostocaceae;g__;s__ | 0.0012 | 0.0025 | 0.0018 | 0.0003 | 0.0000 |
| k__Bacteria;p__Firmicutes;c__Bacilli;o__Lactobacillales;f__Leuconostocaceae;g__Weissella;Other | 0.0343 | 0.1106 | 0.0186 | 0.0035 | 0.0042 |
| k__Bacteria;p__Firmicutes;c__Bacilli;o__Lactobacillales;f__Streptococcaceae;g__Lactococcus;s__ | 0.0018 | 0.0000 | 0.0070 | 0.0000 | 0.0000 |
| k__Bacteria;p__Firmicutes;c__Bacilli;o__Lactobacillales;f__Streptococcaceae;g__Streptococcus;Other | 0.0004 | 0.0003 | 0.0002 | 0.0005 | 0.0005 |
| k__Bacteria;p__Firmicutes;c__Bacilli;o__Lactobacillales;f__Streptococcaceae;g__Streptococcus;s__ | 2.4114 | 3.4098 | 2.5331 | 1.1091 | 2.5938 |
| k__Bacteria;p__Firmicutes;c__Bacilli;o__Lactobacillales;f__Streptococcaceae;g__Streptococcus;s__anginosus | 0.0001 | 0.0000 | 0.0000 | 0.0002 | 0.0000 |
| k__Bacteria;p__Firmicutes;c__Bacilli;o__Lactobacillales;f__Streptococcaceae;g__Streptococcus;s__luteciae | 0.0003 | 0.0009 | 0.0002 | 0.0002 | 0.0000 |
| k__Bacteria;p__Firmicutes;c__Bacilli;o__Lactobacillales;f__Streptococcaceae;g__Streptococcus;s__sobrinus | 0.0001 | 0.0002 | 0.0000 | 0.0000 | 0.0000 |
| k__Bacteria;p__Firmicutes;c__Bacilli;o__Turicibacterales;f__Turicibacteraceae;g__Turicibacter;s__ | 0.2041 | 0.5157 | 0.2371 | 0.0277 | 0.0359 |
| k__Bacteria;p__Firmicutes;c__Clostridia;o__;f__;g__;s__ | 0.0004 | 0.0000 | 0.0000 | 0.0000 | 0.0018 |
| k__Bacteria;p__Firmicutes;c__Clostridia;o__Clostridiales;Other;Other;Other | 0.2422 | 0.1396 | 0.1661 | 0.3311 | 0.3322 |
| k__Bacteria;p__Firmicutes;c__Clostridia;o__Clostridiales;f__;g__;s__ | 8.1656 | 6.5387 | 7.6904 | 8.4689 | 9.9643 |
| k__Bacteria;p__Firmicutes;c__Clostridia;o__Clostridiales;f__Christensenellaceae;g__;s__ | 0.2039 | 0.2952 | 0.2218 | 0.1310 | 0.1677 |
| k__Bacteria;p__Firmicutes;c__Clostridia;o__Clostridiales;f__Clostridiaceae;Other;Other | 0.8655 | 2.0187 | 0.8550 | 0.2289 | 0.3593 |
| k__Bacteria;p__Firmicutes;c__Clostridia;o__Clostridiales;f__Clostridiaceae;g__;s__ | 2.8801 | 6.3316 | 3.4983 | 0.6270 | 1.0635 |
| k__Bacteria;p__Firmicutes;c__Clostridia;o__Clostridiales;f__Clostridiaceae;g__Alkaliphilus;s__ | 0.0001 | 0.0000 | 0.0000 | 0.0003 | 0.0000 |
| k__Bacteria;p__Firmicutes;c__Clostridia;o__Clostridiales;f__Clostridiaceae;g__Clostridium;Other | 0.1502 | 0.4438 | 0.0833 | 0.0255 | 0.0482 |
| k__Bacteria;p__Firmicutes;c__Clostridia;o__Clostridiales;f__Clostridiaceae;g__Clostridium;s__ | 0.2239 | 0.1806 | 0.2332 | 0.2256 | 0.2564 |
| k__Bacteria;p__Firmicutes;c__Clostridia;o__Clostridiales;f__Clostridiaceae;g__Clostridium;s__butyricum | 0.0038 | 0.0140 | 0.0000 | 0.0000 | 0.0012 |
| k__Bacteria;p__Firmicutes;c__Clostridia;o__Clostridiales;f__Clostridiaceae;g__Clostridium;s__neonatale | 0.0001 | 0.0000 | 0.0000 | 0.0000 | 0.0005 |
| k__Bacteria;p__Firmicutes;c__Clostridia;o__Clostridiales;f__Clostridiaceae;g__Clostridium;s__perfringens | 0.0001 | 0.0002 | 0.0000 | 0.0000 | 0.0003 |
| k__Bacteria;p__Firmicutes;c__Clostridia;o__Clostridiales;f__Clostridiaceae;g__Proteiniclasticum;s__ | 0.0002 | 0.0007 | 0.0000 | 0.0000 | 0.0000 |
| k__Bacteria;p__Firmicutes;c__Clostridia;o__Clostridiales;f__Clostridiaceae;g__SMB53;s__ | 0.0017 | 0.0022 | 0.0041 | 0.0000 | 0.0006 |
| k__Bacteria;p__Firmicutes;c__Clostridia;o__Clostridiales;f__Dehalobacteriaceae;g__;s__ | 0.0008 | 0.0000 | 0.0004 | 0.0018 | 0.0009 |
| k__Bacteria;p__Firmicutes;c__Clostridia;o__Clostridiales;f__Dehalobacteriaceae;g__Dehalobacterium;s__ | 0.0062 | 0.0040 | 0.0077 | 0.0083 | 0.0047 |
| k__Bacteria;p__Firmicutes;c__Clostridia;o__Clostridiales;f__Eubacteriaceae;g__Anaerofustis;s__ | 0.0023 | 0.0014 | 0.0000 | 0.0005 | 0.0074 |
| k__Bacteria;p__Firmicutes;c__Clostridia;o__Clostridiales;f__Eubacteriaceae;g__Pseudoramibacter_Eubacterium;s__ | 0.0016 | 0.0065 | 0.0000 | 0.0000 | 0.0000 |
| k__Bacteria;p__Firmicutes;c__Clostridia;o__Clostridiales;f__Lachnospiraceae;Other;Other | 0.7799 | 0.8326 | 0.7466 | 0.7176 | 0.8228 |
| k__Bacteria;p__Firmicutes;c__Clostridia;o__Clostridiales;f__Lachnospiraceae;g__;s__ | 7.3547 | 5.8183 | 6.6100 | 6.0872 | 10.9033 |
| k__Bacteria;p__Firmicutes;c__Clostridia;o__Clostridiales;f__Lachnospiraceae;g__Anaerostipes;s__ | 0.0271 | 0.0309 | 0.0448 | 0.0171 | 0.0155 |
| k__Bacteria;p__Firmicutes;c__Clostridia;o__Clostridiales;f__Lachnospiraceae;g__Blautia;Other | 0.0005 | 0.0004 | 0.0013 | 0.0000 | 0.0003 |
| k__Bacteria;p__Firmicutes;c__Clostridia;o__Clostridiales;f__Lachnospiraceae;g__Blautia;s__ | 1.3752 | 1.6821 | 1.1935 | 1.3384 | 1.2868 |
| k__Bacteria;p__Firmicutes;c__Clostridia;o__Clostridiales;f__Lachnospiraceae;g__Blautia;s__producta | 0.0025 | 0.0002 | 0.0034 | 0.0013 | 0.0050 |
| k__Bacteria;p__Firmicutes;c__Clostridia;o__Clostridiales;f__Lachnospiraceae;g__Butyrivibrio;s__ | 0.0270 | 0.0699 | 0.0301 | 0.0078 | 0.0002 |
| k__Bacteria;p__Firmicutes;c__Clostridia;o__Clostridiales;f__Lachnospiraceae;g__Catonella;s__ | 0.0030 | 0.0065 | 0.0006 | 0.0047 | 0.0000 |
| k__Bacteria;p__Firmicutes;c__Clostridia;o__Clostridiales;f__Lachnospiraceae;g__Coprococcus;Other | 0.0004 | 0.0004 | 0.0000 | 0.0010 | 0.0002 |
| k__Bacteria;p__Firmicutes;c__Clostridia;o__Clostridiales;f__Lachnospiraceae;g__Coprococcus;s__ | 1.6735 | 1.5135 | 1.5004 | 1.9667 | 1.7135 |
| k__Bacteria;p__Firmicutes;c__Clostridia;o__Clostridiales;f__Lachnospiraceae;g__Coprococcus;s__eutactus | 0.0389 | 0.0582 | 0.0359 | 0.0312 | 0.0303 |
| k__Bacteria;p__Firmicutes;c__Clostridia;o__Clostridiales;f__Lachnospiraceae;g__Dorea;s__ | 1.0569 | 1.2172 | 1.0124 | 0.8402 | 1.1577 |
| k__Bacteria;p__Firmicutes;c__Clostridia;o__Clostridiales;f__Lachnospiraceae;g__Dorea;s__formicigenerans | 0.0148 | 0.0381 | 0.0106 | 0.0063 | 0.0040 |
| k__Bacteria;p__Firmicutes;c__Clostridia;o__Clostridiales;f__Lachnospiraceae;g__Epulopiscium;s__ | 0.0081 | 0.0094 | 0.0063 | 0.0049 | 0.0118 |
| k__Bacteria;p__Firmicutes;c__Clostridia;o__Clostridiales;f__Lachnospiraceae;g__Lachnobacterium;s__ | 0.0001 | 0.0000 | 0.0000 | 0.0002 | 0.0003 |
| k__Bacteria;p__Firmicutes;c__Clostridia;o__Clostridiales;f__Lachnospiraceae;g__Lachnospira;s__ | 2.1447 | 1.9062 | 1.3762 | 1.9510 | 3.3454 |
| k__Bacteria;p__Firmicutes;c__Clostridia;o__Clostridiales;f__Lachnospiraceae;g__Moryella;s__ | 0.0003 | 0.0005 | 0.0000 | 0.0002 | 0.0006 |
| k__Bacteria;p__Firmicutes;c__Clostridia;o__Clostridiales;f__Lachnospiraceae;g__Oribacterium;s__ | 0.0010 | 0.0012 | 0.0008 | 0.0003 | 0.0016 |
| k__Bacteria;p__Firmicutes;c__Clostridia;o__Clostridiales;f__Lachnospiraceae;g__Pseudobutyrivibrio;s__ | 0.0008 | 0.0014 | 0.0008 | 0.0000 | 0.0010 |
| k__Bacteria;p__Firmicutes;c__Clostridia;o__Clostridiales;f__Lachnospiraceae;g__Roseburia;Other | 0.0346 | 0.0349 | 0.0276 | 0.0144 | 0.0616 |
| k__Bacteria;p__Firmicutes;c__Clostridia;o__Clostridiales;f__Lachnospiraceae;g__Roseburia;s__ | 0.0378 | 0.0311 | 0.0273 | 0.0273 | 0.0657 |
| k__Bacteria;p__Firmicutes;c__Clostridia;o__Clostridiales;f__Lachnospiraceae;g__Roseburia;s__faecis | 0.0001 | 0.0000 | 0.0000 | 0.0000 | 0.0005 |
| k__Bacteria;p__Firmicutes;c__Clostridia;o__Clostridiales;f__Lachnospiraceae;g__Shuttleworthia;s__ | 0.0186 | 0.0718 | 0.0015 | 0.0000 | 0.0011 |
| k__Bacteria;p__Firmicutes;c__Clostridia;o__Clostridiales;f__Lachnospiraceae;g__[Ruminococcus];Other | 0.0004 | 0.0000 | 0.0006 | 0.0002 | 0.0007 |
| k__Bacteria;p__Firmicutes;c__Clostridia;o__Clostridiales;f__Lachnospiraceae;g__[Ruminococcus];s__ | 0.0578 | 0.1133 | 0.0548 | 0.0291 | 0.0340 |
| k__Bacteria;p__Firmicutes;c__Clostridia;o__Clostridiales;f__Lachnospiraceae;g__[Ruminococcus];s__gnavus | 0.0535 | 0.0813 | 0.0577 | 0.0330 | 0.0422 |
| k__Bacteria;p__Firmicutes;c__Clostridia;o__Clostridiales;f__Peptococcaceae;g__;s__ | 0.0010 | 0.0000 | 0.0000 | 0.0028 | 0.0012 |
| k__Bacteria;p__Firmicutes;c__Clostridia;o__Clostridiales;f__Peptococcaceae;g__Peptococcus;s__ | 0.0281 | 0.0521 | 0.0477 | 0.0087 | 0.0039 |
| k__Bacteria;p__Firmicutes;c__Clostridia;o__Clostridiales;f__Peptococcaceae;g__rc4-4;s__ | 0.0596 | 0.0265 | 0.0578 | 0.0824 | 0.0717 |
| k__Bacteria;p__Firmicutes;c__Clostridia;o__Clostridiales;f__Peptostreptococcaceae;g__;s__ | 0.2363 | 0.3835 | 0.1744 | 0.1841 | 0.2032 |
| k__Bacteria;p__Firmicutes;c__Clostridia;o__Clostridiales;f__Peptostreptococcaceae;g__Filifactor;s__ | 0.0224 | 0.0759 | 0.0128 | 0.0000 | 0.0009 |
| k__Bacteria;p__Firmicutes;c__Clostridia;o__Clostridiales;f__Peptostreptococcaceae;g__Peptostreptococcus;Other | 0.2123 | 0.5501 | 0.2621 | 0.0354 | 0.0015 |
| k__Bacteria;p__Firmicutes;c__Clostridia;o__Clostridiales;f__Peptostreptococcaceae;g__Peptostreptococcus;s__ | 0.0006 | 0.0002 | 0.0021 | 0.0000 | 0.0000 |
| k__Bacteria;p__Firmicutes;c__Clostridia;o__Clostridiales;f__Peptostreptococcaceae;g__Peptostreptococcus;s__anaerobius | 0.0001 | 0.0003 | 0.0002 | 0.0000 | 0.0000 |
| k__Bacteria;p__Firmicutes;c__Clostridia;o__Clostridiales;f__Ruminococcaceae;Other;Other | 0.0029 | 0.0012 | 0.0034 | 0.0038 | 0.0031 |
| k__Bacteria;p__Firmicutes;c__Clostridia;o__Clostridiales;f__Ruminococcaceae;g__;s__ | 11.2648 | 10.5254 | 11.4579 | 10.1162 | 12.9598 |
| k__Bacteria;p__Firmicutes;c__Clostridia;o__Clostridiales;f__Ruminococcaceae;g__Anaerotruncus;s__ | 0.0019 | 0.0004 | 0.0037 | 0.0000 | 0.0034 |
| k__Bacteria;p__Firmicutes;c__Clostridia;o__Clostridiales;f__Ruminococcaceae;g__Faecalibacterium;s__prausnitzii | 2.7716 | 1.9758 | 1.9776 | 3.0544 | 4.0786 |
| k__Bacteria;p__Firmicutes;c__Clostridia;o__Clostridiales;f__Ruminococcaceae;g__Oscillospira;s__ | 1.5922 | 1.5182 | 1.8346 | 1.1789 | 1.8369 |
| k__Bacteria;p__Firmicutes;c__Clostridia;o__Clostridiales;f__Ruminococcaceae;g__Ruminococcus;Other | 0.1362 | 0.1913 | 0.1833 | 0.0523 | 0.1180 |
| k__Bacteria;p__Firmicutes;c__Clostridia;o__Clostridiales;f__Ruminococcaceae;g__Ruminococcus;s__ | 1.6050 | 1.5532 | 1.6110 | 1.7344 | 1.5215 |
| k__Bacteria;p__Firmicutes;c__Clostridia;o__Clostridiales;f__Ruminococcaceae;g__Ruminococcus;s__bromii | 0.0002 | 0.0000 | 0.0006 | 0.0000 | 0.0000 |
| k__Bacteria;p__Firmicutes;c__Clostridia;o__Clostridiales;f__Ruminococcaceae;g__Ruminococcus;s__flavefaciens | 0.3518 | 0.3193 | 0.4242 | 0.3996 | 0.2640 |
| k__Bacteria;p__Firmicutes;c__Clostridia;o__Clostridiales;f__Symbiobacteriaceae;g__Symbiobacterium;s__ | 0.0002 | 0.0000 | 0.0006 | 0.0000 | 0.0000 |
| k__Bacteria;p__Firmicutes;c__Clostridia;o__Clostridiales;f__Syntrophomonadaceae;g__Syntrophomonas;s__ | 0.0010 | 0.0024 | 0.0012 | 0.0000 | 0.0003 |
| k__Bacteria;p__Firmicutes;c__Clostridia;o__Clostridiales;f__Veillonellaceae;Other;Other | 0.0002 | 0.0000 | 0.0005 | 0.0002 | 0.0003 |
| k__Bacteria;p__Firmicutes;c__Clostridia;o__Clostridiales;f__Veillonellaceae;g__;s__ | 0.0308 | 0.0328 | 0.0399 | 0.0232 | 0.0273 |
| k__Bacteria;p__Firmicutes;c__Clostridia;o__Clostridiales;f__Veillonellaceae;g__Acidaminococcus;s__ | 0.0120 | 0.0436 | 0.0015 | 0.0029 | 0.0000 |
| k__Bacteria;p__Firmicutes;c__Clostridia;o__Clostridiales;f__Veillonellaceae;g__Anaerovibrio;s__ | 0.2773 | 0.1808 | 0.2690 | 0.3704 | 0.2890 |
| k__Bacteria;p__Firmicutes;c__Clostridia;o__Clostridiales;f__Veillonellaceae;g__Dialister;s__ | 0.0819 | 0.1901 | 0.1043 | 0.0298 | 0.0034 |
| k__Bacteria;p__Firmicutes;c__Clostridia;o__Clostridiales;f__Veillonellaceae;g__Megasphaera;s__ | 0.2729 | 0.7378 | 0.2312 | 0.0733 | 0.0494 |
| k__Bacteria;p__Firmicutes;c__Clostridia;o__Clostridiales;f__Veillonellaceae;g__Mitsuokella;Other | 0.0011 | 0.0014 | 0.0011 | 0.0012 | 0.0005 |
| k__Bacteria;p__Firmicutes;c__Clostridia;o__Clostridiales;f__Veillonellaceae;g__Mitsuokella;s__ | 0.0175 | 0.0298 | 0.0203 | 0.0081 | 0.0117 |
| k__Bacteria;p__Firmicutes;c__Clostridia;o__Clostridiales;f__Veillonellaceae;g__Mitsuokella;s__multacida | 0.0392 | 0.0599 | 0.0197 | 0.0415 | 0.0357 |
| k__Bacteria;p__Firmicutes;c__Clostridia;o__Clostridiales;f__Veillonellaceae;g__Phascolarctobacterium;s__ | 0.6680 | 0.5210 | 0.4857 | 0.8320 | 0.8334 |
| k__Bacteria;p__Firmicutes;c__Clostridia;o__Clostridiales;f__Veillonellaceae;g__Selenomonas;Other | 0.0001 | 0.0002 | 0.0000 | 0.0000 | 0.0000 |
| k__Bacteria;p__Firmicutes;c__Clostridia;o__Clostridiales;f__Veillonellaceae;g__Selenomonas;s__ | 0.0022 | 0.0008 | 0.0080 | 0.0000 | 0.0000 |
| k__Bacteria;p__Firmicutes;c__Clostridia;o__Clostridiales;f__Veillonellaceae;g__Veillonella;s__ | 0.0005 | 0.0007 | 0.0000 | 0.0000 | 0.0012 |
| k__Bacteria;p__Firmicutes;c__Clostridia;o__Clostridiales;f__Veillonellaceae;g__Veillonella;s__dispar | 0.0151 | 0.0262 | 0.0127 | 0.0117 | 0.0099 |
| k__Bacteria;p__Firmicutes;c__Clostridia;o__Clostridiales;f__[Mogibacteriaceae];g__;s__ | 0.4125 | 0.5289 | 0.4301 | 0.3131 | 0.3780 |
| k__Bacteria;p__Firmicutes;c__Clostridia;o__Clostridiales;f__[Mogibacteriaceae];g__Anaerovorax;s__ | 0.0051 | 0.0000 | 0.0080 | 0.0019 | 0.0104 |
| k__Bacteria;p__Firmicutes;c__Clostridia;o__Clostridiales;f__[Mogibacteriaceae];g__Mogibacterium;s__ | 0.0291 | 0.0127 | 0.0233 | 0.0570 | 0.0235 |
| k__Bacteria;p__Firmicutes;c__Clostridia;o__Clostridiales;f__[Tissierellaceae];g__Anaerococcus;s__ | 0.0503 | 0.0685 | 0.1078 | 0.0039 | 0.0208 |
| k__Bacteria;p__Firmicutes;c__Clostridia;o__Clostridiales;f__[Tissierellaceae];g__Finegoldia;s__ | 0.0124 | 0.0406 | 0.0090 | 0.0000 | 0.0000 |
| k__Bacteria;p__Firmicutes;c__Clostridia;o__Clostridiales;f__[Tissierellaceae];g__Gallicola;s__ | 0.0002 | 0.0000 | 0.0000 | 0.0000 | 0.0009 |
| k__Bacteria;p__Firmicutes;c__Clostridia;o__Clostridiales;f__[Tissierellaceae];g__Helcococcus;s__ | 0.0504 | 0.1379 | 0.0593 | 0.0026 | 0.0018 |
| k__Bacteria;p__Firmicutes;c__Clostridia;o__Clostridiales;f__[Tissierellaceae];g__Parvimonas;s__ | 0.0974 | 0.3085 | 0.0652 | 0.0157 | 0.0000 |
| k__Bacteria;p__Firmicutes;c__Clostridia;o__Clostridiales;f__[Tissierellaceae];g__Peptoniphilus;s__ | 0.0157 | 0.0311 | 0.0265 | 0.0009 | 0.0041 |
| k__Bacteria;p__Firmicutes;c__Clostridia;o__Clostridiales;f__[Tissierellaceae];g__ph2;s__ | 0.0088 | 0.0236 | 0.0117 | 0.0000 | 0.0000 |
| k__Bacteria;p__Firmicutes;c__Erysipelotrichi;o__Erysipelotrichales;f__Erysipelotrichaceae;Other;Other | 0.0001 | 0.0000 | 0.0000 | 0.0005 | 0.0000 |
| k__Bacteria;p__Firmicutes;c__Erysipelotrichi;o__Erysipelotrichales;f__Erysipelotrichaceae;g__;s__ | 0.5176 | 0.7977 | 0.4090 | 0.3435 | 0.5203 |
| k__Bacteria;p__Firmicutes;c__Erysipelotrichi;o__Erysipelotrichales;f__Erysipelotrichaceae;g__Asteroleplasma;s__anaerobium | 0.0002 | 0.0000 | 0.0008 | 0.0000 | 0.0000 |
| k__Bacteria;p__Firmicutes;c__Erysipelotrichi;o__Erysipelotrichales;f__Erysipelotrichaceae;g__Bulleidia;Other | 0.0000 | 0.0002 | 0.0000 | 0.0000 | 0.0000 |
| k__Bacteria;p__Firmicutes;c__Erysipelotrichi;o__Erysipelotrichales;f__Erysipelotrichaceae;g__Bulleidia;s__ | 0.0494 | 0.1797 | 0.0145 | 0.0023 | 0.0012 |
| k__Bacteria;p__Firmicutes;c__Erysipelotrichi;o__Erysipelotrichales;f__Erysipelotrichaceae;g__Bulleidia;s__p-1630-c5 | 0.3910 | 0.4447 | 0.4514 | 0.3233 | 0.3448 |
| k__Bacteria;p__Firmicutes;c__Erysipelotrichi;o__Erysipelotrichales;f__Erysipelotrichaceae;g__Catenibacterium;s__ | 0.3236 | 0.4621 | 0.2370 | 0.3125 | 0.2827 |
| k__Bacteria;p__Firmicutes;c__Erysipelotrichi;o__Erysipelotrichales;f__Erysipelotrichaceae;g__Coprobacillus;s__ | 0.0477 | 0.0201 | 0.0603 | 0.0173 | 0.0931 |
| k__Bacteria;p__Firmicutes;c__Erysipelotrichi;o__Erysipelotrichales;f__Erysipelotrichaceae;g__L7A_E11;s__ | 0.0122 | 0.0024 | 0.0066 | 0.0095 | 0.0304 |
| k__Bacteria;p__Firmicutes;c__Erysipelotrichi;o__Erysipelotrichales;f__Erysipelotrichaceae;g__PSB-M-3;s__ | 0.0242 | 0.0079 | 0.0150 | 0.0210 | 0.0526 |
| k__Bacteria;p__Firmicutes;c__Erysipelotrichi;o__Erysipelotrichales;f__Erysipelotrichaceae;g__RFN20;s__ | 0.0442 | 0.0540 | 0.0240 | 0.0414 | 0.0574 |
| k__Bacteria;p__Firmicutes;c__Erysipelotrichi;o__Erysipelotrichales;f__Erysipelotrichaceae;g__Sharpea;Other | 0.0352 | 0.0384 | 0.0340 | 0.0526 | 0.0159 |
| k__Bacteria;p__Firmicutes;c__Erysipelotrichi;o__Erysipelotrichales;f__Erysipelotrichaceae;g__Sharpea;s__azabuensis | 0.0001 | 0.0000 | 0.0002 | 0.0000 | 0.0000 |
| k__Bacteria;p__Firmicutes;c__Erysipelotrichi;o__Erysipelotrichales;f__Erysipelotrichaceae;g__Sharpea;s__p-3329-23G2 | 0.0030 | 0.0116 | 0.0000 | 0.0000 | 0.0006 |
| k__Bacteria;p__Firmicutes;c__Erysipelotrichi;o__Erysipelotrichales;f__Erysipelotrichaceae;g__[Eubacterium];s__biforme | 0.3608 | 0.7336 | 0.2523 | 0.2288 | 0.2287 |
| k__Bacteria;p__Firmicutes;c__Erysipelotrichi;o__Erysipelotrichales;f__Erysipelotrichaceae;g__[Eubacterium];s__cylindroides | 0.0162 | 0.0551 | 0.0058 | 0.0009 | 0.0029 |
| k__Bacteria;p__Firmicutes;c__Erysipelotrichi;o__Erysipelotrichales;f__Erysipelotrichaceae;g__p-75-a5;s__ | 0.5093 | 0.3780 | 0.4917 | 0.4500 | 0.7176 |
| k__Bacteria;p__Fusobacteria;c__Fusobacteriia;o__Fusobacteriales;f__Fusobacteriaceae;g__Fusobacterium;s__ | 0.2077 | 0.5333 | 0.2124 | 0.0676 | 0.0175 |
| k__Bacteria;p__Fusobacteria;c__Fusobacteriia;o__Fusobacteriales;f__Leptotrichiaceae;g__Leptotrichia;s__ | 0.0068 | 0.0051 | 0.0025 | 0.0092 | 0.0103 |
| k__Bacteria;p__GN02;c__BD1-5;o__;f__;g__;s__ | 0.0005 | 0.0000 | 0.0010 | 0.0009 | 0.0000 |
| k__Bacteria;p__Gemmatimonadetes;c__Gemmatimonadetes;o__Gemmatimonadales;f__Ellin5301;g__;s__ | 0.0002 | 0.0008 | 0.0000 | 0.0000 | 0.0000 |
| k__Bacteria;p__Lentisphaerae;c__[Lentisphaeria];o__Victivallales;f__Victivallaceae;g__;s__ | 0.0019 | 0.0004 | 0.0027 | 0.0000 | 0.0046 |
| k__Bacteria;p__Lentisphaerae;c__[Lentisphaeria];o__Z20;f__R4-45B;g__;s__ | 0.0052 | 0.0066 | 0.0066 | 0.0009 | 0.0065 |
| k__Bacteria;p__Planctomycetes;c__Planctomycetia;o__Pirellulales;f__Pirellulaceae;g__;s__ | 0.0007 | 0.0012 | 0.0000 | 0.0010 | 0.0006 |
| k__Bacteria;p__Proteobacteria;c__Alphaproteobacteria;o__;f__;g__;s__ | 0.0463 | 0.0280 | 0.0309 | 0.0504 | 0.0760 |
| k__Bacteria;p__Proteobacteria;c__Alphaproteobacteria;o__BD7-3;f__;g__;s__ | 0.0011 | 0.0000 | 0.0044 | 0.0000 | 0.0000 |
| k__Bacteria;p__Proteobacteria;c__Alphaproteobacteria;o__Caulobacterales;f__Caulobacteraceae;g__;s__ | 0.0770 | 0.0273 | 0.1227 | 0.1455 | 0.0126 |
| k__Bacteria;p__Proteobacteria;c__Alphaproteobacteria;o__Caulobacterales;f__Caulobacteraceae;g__Brevundimonas;s__diminuta | 0.0030 | 0.0000 | 0.0117 | 0.0003 | 0.0000 |
| k__Bacteria;p__Proteobacteria;c__Alphaproteobacteria;o__Caulobacterales;f__Caulobacteraceae;g__Mycoplana;s__ | 0.0034 | 0.0000 | 0.0002 | 0.0132 | 0.0002 |
| k__Bacteria;p__Proteobacteria;c__Alphaproteobacteria;o__RF32;f__;g__;s__ | 0.6100 | 0.5058 | 0.5382 | 0.6300 | 0.7661 |
| k__Bacteria;p__Proteobacteria;c__Alphaproteobacteria;o__Rhizobiales;f__;g__;s__ | 0.0031 | 0.0000 | 0.0034 | 0.0090 | 0.0000 |
| k__Bacteria;p__Proteobacteria;c__Alphaproteobacteria;o__Rhizobiales;f__Aurantimonadaceae;g__;s__ | 0.0012 | 0.0000 | 0.0002 | 0.0044 | 0.0000 |
| k__Bacteria;p__Proteobacteria;c__Alphaproteobacteria;o__Rhizobiales;f__Bradyrhizobiaceae;Other;Other | 0.0005 | 0.0000 | 0.0007 | 0.0000 | 0.0013 |
| k__Bacteria;p__Proteobacteria;c__Alphaproteobacteria;o__Rhizobiales;f__Bradyrhizobiaceae;g__;s__ | 0.0179 | 0.0174 | 0.0331 | 0.0060 | 0.0149 |
| k__Bacteria;p__Proteobacteria;c__Alphaproteobacteria;o__Rhizobiales;f__Bradyrhizobiaceae;g__Bradyrhizobium;Other | 0.0001 | 0.0000 | 0.0002 | 0.0000 | 0.0000 |
| k__Bacteria;p__Proteobacteria;c__Alphaproteobacteria;o__Rhizobiales;f__Bradyrhizobiaceae;g__Bradyrhizobium;s__ | 0.0001 | 0.0000 | 0.0000 | 0.0000 | 0.0002 |
| k__Bacteria;p__Proteobacteria;c__Alphaproteobacteria;o__Rhizobiales;f__Brucellaceae;g__Ochrobactrum;s__ | 0.0022 | 0.0087 | 0.0000 | 0.0000 | 0.0000 |
| k__Bacteria;p__Proteobacteria;c__Alphaproteobacteria;o__Rhizobiales;f__Hyphomicrobiaceae;g__Devosia;s__ | 0.0012 | 0.0005 | 0.0020 | 0.0002 | 0.0020 |
| k__Bacteria;p__Proteobacteria;c__Alphaproteobacteria;o__Rhizobiales;f__Hyphomicrobiaceae;g__Hyphomicrobium;s__ | 0.0007 | 0.0000 | 0.0029 | 0.0000 | 0.0000 |
| k__Bacteria;p__Proteobacteria;c__Alphaproteobacteria;o__Rhizobiales;f__Hyphomicrobiaceae;g__Rhodoplanes;s__ | 0.0006 | 0.0026 | 0.0000 | 0.0000 | 0.0000 |
| k__Bacteria;p__Proteobacteria;c__Alphaproteobacteria;o__Rhizobiales;f__Methylobacteriaceae;g__;s__ | 0.0003 | 0.0000 | 0.0000 | 0.0012 | 0.0000 |
| k__Bacteria;p__Proteobacteria;c__Alphaproteobacteria;o__Rhizobiales;f__Methylobacteriaceae;g__Methylobacterium;s__ | 0.0032 | 0.0000 | 0.0078 | 0.0000 | 0.0050 |
| k__Bacteria;p__Proteobacteria;c__Alphaproteobacteria;o__Rhizobiales;f__Methylobacteriaceae;g__Methylobacterium;s__adhaesivum | 0.0103 | 0.0379 | 0.0006 | 0.0027 | 0.0000 |
| k__Bacteria;p__Proteobacteria;c__Alphaproteobacteria;o__Rhizobiales;f__Methylocystaceae;g__;s__ | 0.0015 | 0.0000 | 0.0000 | 0.0061 | 0.0000 |
| k__Bacteria;p__Proteobacteria;c__Alphaproteobacteria;o__Rhizobiales;f__Phyllobacteriaceae;g__;s__ | 0.0042 | 0.0028 | 0.0083 | 0.0045 | 0.0012 |
| k__Bacteria;p__Proteobacteria;c__Alphaproteobacteria;o__Rhizobiales;f__Rhizobiaceae;g__Agrobacterium;s__ | 0.0031 | 0.0002 | 0.0064 | 0.0046 | 0.0012 |
| k__Bacteria;p__Proteobacteria;c__Alphaproteobacteria;o__Rhizobiales;f__Rhizobiaceae;g__Kaistia;s__ | 0.0005 | 0.0000 | 0.0020 | 0.0000 | 0.0000 |
| k__Bacteria;p__Proteobacteria;c__Alphaproteobacteria;o__Rhodobacterales;f__Hyphomonadaceae;g__;s__ | 0.0013 | 0.0000 | 0.0000 | 0.0000 | 0.0054 |
| k__Bacteria;p__Proteobacteria;c__Alphaproteobacteria;o__Rhodobacterales;f__Rhodobacteraceae;Other;Other | 0.0002 | 0.0000 | 0.0007 | 0.0000 | 0.0000 |
| k__Bacteria;p__Proteobacteria;c__Alphaproteobacteria;o__Rhodobacterales;f__Rhodobacteraceae;g__;s__ | 0.0006 | 0.0005 | 0.0018 | 0.0003 | 0.0000 |
| k__Bacteria;p__Proteobacteria;c__Alphaproteobacteria;o__Rhodobacterales;f__Rhodobacteraceae;g__Paracoccus;Other | 0.0012 | 0.0003 | 0.0020 | 0.0024 | 0.0000 |
| k__Bacteria;p__Proteobacteria;c__Alphaproteobacteria;o__Rhodobacterales;f__Rhodobacteraceae;g__Paracoccus;s__ | 0.2199 | 0.0707 | 0.4118 | 0.3615 | 0.0356 |
| k__Bacteria;p__Proteobacteria;c__Alphaproteobacteria;o__Rhodobacterales;f__Rhodobacteraceae;g__Paracoccus;s__aminovorans | 0.0470 | 0.0383 | 0.1132 | 0.0357 | 0.0006 |
| k__Bacteria;p__Proteobacteria;c__Alphaproteobacteria;o__Rhodobacterales;f__Rhodobacteraceae;g__Paracoccus;s__marcusii | 0.1806 | 0.0957 | 0.3698 | 0.2473 | 0.0097 |
| k__Bacteria;p__Proteobacteria;c__Alphaproteobacteria;o__Rhodobacterales;f__Rhodobacteraceae;g__Rhodobacter;s__ | 0.0001 | 0.0000 | 0.0000 | 0.0000 | 0.0006 |
| k__Bacteria;p__Proteobacteria;c__Alphaproteobacteria;o__Rhodobacterales;f__Rhodobacteraceae;g__Rubellimicrobium;s__ | 0.0009 | 0.0030 | 0.0006 | 0.0000 | 0.0000 |
| k__Bacteria;p__Proteobacteria;c__Alphaproteobacteria;o__Rhodospirillales;f__;g__;s__ | 0.0012 | 0.0000 | 0.0046 | 0.0000 | 0.0000 |
| k__Bacteria;p__Proteobacteria;c__Alphaproteobacteria;o__Rhodospirillales;f__Acetobacteraceae;g__;s__ | 0.0022 | 0.0004 | 0.0085 | 0.0000 | 0.0000 |
| k__Bacteria;p__Proteobacteria;c__Alphaproteobacteria;o__Rhodospirillales;f__Rhodospirillaceae;g__;s__ | 0.0012 | 0.0000 | 0.0049 | 0.0000 | 0.0000 |
| k__Bacteria;p__Proteobacteria;c__Alphaproteobacteria;o__Rickettsiales;f__;g__;s__ | 0.0008 | 0.0022 | 0.0000 | 0.0000 | 0.0009 |
| k__Bacteria;p__Proteobacteria;c__Alphaproteobacteria;o__Rickettsiales;f__mitochondria;Other;Other | 0.0143 | 0.0321 | 0.0212 | 0.0016 | 0.0022 |
| k__Bacteria;p__Proteobacteria;c__Alphaproteobacteria;o__Sphingomonadales;Other;Other;Other | 0.0003 | 0.0000 | 0.0005 | 0.0005 | 0.0000 |
| k__Bacteria;p__Proteobacteria;c__Alphaproteobacteria;o__Sphingomonadales;f__Erythrobacteraceae;g__;s__ | 0.0260 | 0.0130 | 0.0784 | 0.0127 | 0.0000 |
| k__Bacteria;p__Proteobacteria;c__Alphaproteobacteria;o__Sphingomonadales;f__Sphingomonadaceae;Other;Other | 0.0589 | 0.0139 | 0.1053 | 0.1122 | 0.0043 |
| k__Bacteria;p__Proteobacteria;c__Alphaproteobacteria;o__Sphingomonadales;f__Sphingomonadaceae;g__;s__ | 0.0089 | 0.0024 | 0.0170 | 0.0164 | 0.0000 |
| k__Bacteria;p__Proteobacteria;c__Alphaproteobacteria;o__Sphingomonadales;f__Sphingomonadaceae;g__Kaistobacter;s__ | 0.0001 | 0.0000 | 0.0000 | 0.0006 | 0.0000 |
| k__Bacteria;p__Proteobacteria;c__Alphaproteobacteria;o__Sphingomonadales;f__Sphingomonadaceae;g__Novosphingobium;s__ | 0.0098 | 0.0027 | 0.0068 | 0.0291 | 0.0007 |
| k__Bacteria;p__Proteobacteria;c__Alphaproteobacteria;o__Sphingomonadales;f__Sphingomonadaceae;g__Sphingobium;s__ | 0.0057 | 0.0088 | 0.0100 | 0.0040 | 0.0000 |
| k__Bacteria;p__Proteobacteria;c__Alphaproteobacteria;o__Sphingomonadales;f__Sphingomonadaceae;g__Sphingomonas;Other | 0.0039 | 0.0043 | 0.0071 | 0.0040 | 0.0000 |
| k__Bacteria;p__Proteobacteria;c__Alphaproteobacteria;o__Sphingomonadales;f__Sphingomonadaceae;g__Sphingomonas;s__ | 0.0836 | 0.0323 | 0.1993 | 0.0996 | 0.0032 |
| k__Bacteria;p__Proteobacteria;c__Alphaproteobacteria;o__Sphingomonadales;f__Sphingomonadaceae;g__Sphingomonas;s__wittichii | 0.0006 | 0.0000 | 0.0007 | 0.0018 | 0.0000 |
| k__Bacteria;p__Proteobacteria;c__Alphaproteobacteria;o__Sphingomonadales;f__Sphingomonadaceae;g__Sphingomonas;s__yabuuchiae | 0.0001 | 0.0000 | 0.0002 | 0.0003 | 0.0000 |
| k__Bacteria;p__Proteobacteria;c__Betaproteobacteria;Other;Other;Other;Other | 0.0001 | 0.0000 | 0.0000 | 0.0000 | 0.0006 |
| k__Bacteria;p__Proteobacteria;c__Betaproteobacteria;o__ASSO-13;f__;g__;s__ | 0.0001 | 0.0000 | 0.0000 | 0.0000 | 0.0003 |
| k__Bacteria;p__Proteobacteria;c__Betaproteobacteria;o__Burkholderiales;f__Alcaligenaceae;g__;s__ | 0.0029 | 0.0002 | 0.0056 | 0.0027 | 0.0032 |
| k__Bacteria;p__Proteobacteria;c__Betaproteobacteria;o__Burkholderiales;f__Alcaligenaceae;g__Achromobacter;s__ | 0.0038 | 0.0035 | 0.0048 | 0.0012 | 0.0056 |
| k__Bacteria;p__Proteobacteria;c__Betaproteobacteria;o__Burkholderiales;f__Alcaligenaceae;g__Oligella;s__ | 0.0008 | 0.0033 | 0.0000 | 0.0000 | 0.0000 |
| k__Bacteria;p__Proteobacteria;c__Betaproteobacteria;o__Burkholderiales;f__Alcaligenaceae;g__Sutterella;s__ | 0.2099 | 0.2126 | 0.1418 | 0.2687 | 0.2164 |
| k__Bacteria;p__Proteobacteria;c__Betaproteobacteria;o__Burkholderiales;f__Burkholderiaceae;g__Lautropia;s__ | 0.0090 | 0.0296 | 0.0042 | 0.0023 | 0.0000 |
| k__Bacteria;p__Proteobacteria;c__Betaproteobacteria;o__Burkholderiales;f__Comamonadaceae;Other;Other | 0.0022 | 0.0002 | 0.0039 | 0.0045 | 0.0000 |
| k__Bacteria;p__Proteobacteria;c__Betaproteobacteria;o__Burkholderiales;f__Comamonadaceae;g__;s__ | 0.0736 | 0.1533 | 0.0829 | 0.0451 | 0.0129 |
| k__Bacteria;p__Proteobacteria;c__Betaproteobacteria;o__Burkholderiales;f__Comamonadaceae;g__Acidovorax;Other | 0.0001 | 0.0000 | 0.0002 | 0.0003 | 0.0000 |
| k__Bacteria;p__Proteobacteria;c__Betaproteobacteria;o__Burkholderiales;f__Comamonadaceae;g__Comamonas;s__ | 0.0233 | 0.0000 | 0.0102 | 0.0814 | 0.0016 |
| k__Bacteria;p__Proteobacteria;c__Betaproteobacteria;o__Burkholderiales;f__Comamonadaceae;g__Delftia;s__ | 0.0001 | 0.0000 | 0.0000 | 0.0000 | 0.0005 |
| k__Bacteria;p__Proteobacteria;c__Betaproteobacteria;o__Burkholderiales;f__Comamonadaceae;g__Hydrogenophaga;s__ | 0.0004 | 0.0016 | 0.0000 | 0.0000 | 0.0000 |
| k__Bacteria;p__Proteobacteria;c__Betaproteobacteria;o__Burkholderiales;f__Comamonadaceae;g__Limnohabitans;s__ | 0.0004 | 0.0000 | 0.0012 | 0.0002 | 0.0000 |
| k__Bacteria;p__Proteobacteria;c__Betaproteobacteria;o__Burkholderiales;f__Comamonadaceae;g__Variovorax;s__ | 0.0003 | 0.0002 | 0.0000 | 0.0000 | 0.0011 |
| k__Bacteria;p__Proteobacteria;c__Betaproteobacteria;o__Burkholderiales;f__Oxalobacteraceae;g__;s__ | 0.0188 | 0.0134 | 0.0204 | 0.0400 | 0.0012 |
| k__Bacteria;p__Proteobacteria;c__Betaproteobacteria;o__Burkholderiales;f__Oxalobacteraceae;g__Janthinobacterium;s__ | 0.0003 | 0.0000 | 0.0012 | 0.0000 | 0.0000 |
| k__Bacteria;p__Proteobacteria;c__Betaproteobacteria;o__Burkholderiales;f__Oxalobacteraceae;g__Oxalobacter;s__formigenes | 0.0224 | 0.0204 | 0.0181 | 0.0124 | 0.0388 |
| k__Bacteria;p__Proteobacteria;c__Betaproteobacteria;o__Burkholderiales;f__Oxalobacteraceae;g__Ralstonia;s__ | 0.0164 | 0.0379 | 0.0176 | 0.0018 | 0.0082 |
| k__Bacteria;p__Proteobacteria;c__Betaproteobacteria;o__Methylophilales;f__Methylophilaceae;g__Methylotenera;s__mobilis | 0.0005 | 0.0000 | 0.0010 | 0.0000 | 0.0010 |
| k__Bacteria;p__Proteobacteria;c__Betaproteobacteria;o__Neisseriales;f__Neisseriaceae;Other;Other | 0.0125 | 0.0400 | 0.0039 | 0.0057 | 0.0002 |
| k__Bacteria;p__Proteobacteria;c__Betaproteobacteria;o__Neisseriales;f__Neisseriaceae;g__;s__ | 0.0396 | 0.0492 | 0.0402 | 0.0557 | 0.0131 |
| k__Bacteria;p__Proteobacteria;c__Betaproteobacteria;o__Neisseriales;f__Neisseriaceae;g__Eikenella;s__ | 0.0001 | 0.0000 | 0.0005 | 0.0000 | 0.0000 |
| k__Bacteria;p__Proteobacteria;c__Betaproteobacteria;o__Neisseriales;f__Neisseriaceae;g__Kingella;s__ | 0.0009 | 0.0000 | 0.0037 | 0.0000 | 0.0000 |
| k__Bacteria;p__Proteobacteria;c__Betaproteobacteria;o__Neisseriales;f__Neisseriaceae;g__Neisseria;Other | 0.0078 | 0.0245 | 0.0048 | 0.0012 | 0.0006 |
| k__Bacteria;p__Proteobacteria;c__Betaproteobacteria;o__Neisseriales;f__Neisseriaceae;g__Neisseria;s__ | 0.0031 | 0.0118 | 0.0002 | 0.0000 | 0.0005 |
| k__Bacteria;p__Proteobacteria;c__Betaproteobacteria;o__Neisseriales;f__Neisseriaceae;g__Neisseria;s__cinerea | 0.0001 | 0.0000 | 0.0002 | 0.0000 | 0.0000 |
| k__Bacteria;p__Proteobacteria;c__Betaproteobacteria;o__Neisseriales;f__Neisseriaceae;g__Neisseria;s__subflava | 0.0017 | 0.0008 | 0.0035 | 0.0019 | 0.0005 |
| k__Bacteria;p__Proteobacteria;c__Betaproteobacteria;o__Rhodocyclales;f__Rhodocyclaceae;g__Dechloromonas;s__ | 0.0026 | 0.0000 | 0.0000 | 0.0000 | 0.0105 |
| k__Bacteria;p__Proteobacteria;c__Betaproteobacteria;o__Tremblayales;f__;g__;s__ | 0.1759 | 0.1210 | 0.1234 | 0.2389 | 0.2202 |
| k__Bacteria;p__Proteobacteria;c__Deltaproteobacteria;o__Bdellovibrionales;f__Bacteriovoracaceae;g__;s__ | 0.0002 | 0.0000 | 0.0007 | 0.0000 | 0.0000 |
| k__Bacteria;p__Proteobacteria;c__Deltaproteobacteria;o__Bdellovibrionales;f__Bdellovibrionaceae;g__Bdellovibrio;s__ | 0.0002 | 0.0000 | 0.0007 | 0.0000 | 0.0000 |
| k__Bacteria;p__Proteobacteria;c__Deltaproteobacteria;o__Desulfovibrionales;f__Desulfovibrionaceae;g__;s__ | 0.0309 | 0.0309 | 0.0176 | 0.0389 | 0.0361 |
| k__Bacteria;p__Proteobacteria;c__Deltaproteobacteria;o__Desulfovibrionales;f__Desulfovibrionaceae;g__Bilophila;s__ | 0.0040 | 0.0021 | 0.0072 | 0.0009 | 0.0056 |
| k__Bacteria;p__Proteobacteria;c__Deltaproteobacteria;o__Desulfovibrionales;f__Desulfovibrionaceae;g__Desulfovibrio;s__ | 0.1194 | 0.0768 | 0.1259 | 0.0957 | 0.1792 |
| k__Bacteria;p__Proteobacteria;c__Deltaproteobacteria;o__GMD14H09;f__;g__;s__ | 0.0466 | 0.0675 | 0.0889 | 0.0095 | 0.0203 |
| k__Bacteria;p__Proteobacteria;c__Deltaproteobacteria;o__Myxococcales;f__;g__;s__ | 0.0016 | 0.0054 | 0.0000 | 0.0012 | 0.0000 |
| k__Bacteria;p__Proteobacteria;c__Deltaproteobacteria;o__Myxococcales;f__0319-6G20;g__;s__ | 0.0020 | 0.0000 | 0.0000 | 0.0061 | 0.0018 |
| k__Bacteria;p__Proteobacteria;c__Deltaproteobacteria;o__Myxococcales;f__Haliangiaceae;g__;s__ | 0.0001 | 0.0000 | 0.0006 | 0.0000 | 0.0000 |
| k__Bacteria;p__Proteobacteria;c__Epsilonproteobacteria;o__Campylobacterales;f__Campylobacteraceae;g__Arcobacter;s__ | 0.0005 | 0.0000 | 0.0000 | 0.0000 | 0.0018 |
| k__Bacteria;p__Proteobacteria;c__Epsilonproteobacteria;o__Campylobacterales;f__Campylobacteraceae;g__Campylobacter;s__ | 0.7781 | 0.6560 | 0.5990 | 0.7578 | 1.0998 |
| k__Bacteria;p__Proteobacteria;c__Epsilonproteobacteria;o__Campylobacterales;f__Helicobacteraceae;Other;Other | 0.0002 | 0.0000 | 0.0000 | 0.0000 | 0.0006 |
| k__Bacteria;p__Proteobacteria;c__Epsilonproteobacteria;o__Campylobacterales;f__Helicobacteraceae;g__;s__ | 0.1708 | 0.1821 | 0.1637 | 0.0748 | 0.2626 |
| k__Bacteria;p__Proteobacteria;c__Epsilonproteobacteria;o__Campylobacterales;f__Helicobacteraceae;g__Flexispira;s__ | 0.0031 | 0.0030 | 0.0031 | 0.0023 | 0.0041 |
| k__Bacteria;p__Proteobacteria;c__Epsilonproteobacteria;o__Campylobacterales;f__Helicobacteraceae;g__Flexispira;s__rappini | 0.0001 | 0.0000 | 0.0000 | 0.0000 | 0.0003 |
| k__Bacteria;p__Proteobacteria;c__Epsilonproteobacteria;o__Campylobacterales;f__Helicobacteraceae;g__Helicobacter;Other | 0.0069 | 0.0053 | 0.0079 | 0.0047 | 0.0095 |
| k__Bacteria;p__Proteobacteria;c__Epsilonproteobacteria;o__Campylobacterales;f__Helicobacteraceae;g__Helicobacter;s__pylori | 0.0004 | 0.0002 | 0.0005 | 0.0002 | 0.0007 |
| k__Bacteria;p__Proteobacteria;c__Gammaproteobacteria;Other;Other;Other;Other | 0.0001 | 0.0000 | 0.0002 | 0.0000 | 0.0000 |
| k__Bacteria;p__Proteobacteria;c__Gammaproteobacteria;o__Aeromonadales;f__Succinivibrionaceae;g__;s__ | 0.1962 | 0.7609 | 0.0218 | 0.0021 | 0.0000 |
| k__Bacteria;p__Proteobacteria;c__Gammaproteobacteria;o__Aeromonadales;f__Succinivibrionaceae;g__Anaerobiospirillum;s__ | 0.0009 | 0.0002 | 0.0032 | 0.0000 | 0.0000 |
| k__Bacteria;p__Proteobacteria;c__Gammaproteobacteria;o__Aeromonadales;f__Succinivibrionaceae;g__Succinivibrio;s__ | 0.1186 | 0.2338 | 0.0664 | 0.0499 | 0.1244 |
| k__Bacteria;p__Proteobacteria;c__Gammaproteobacteria;o__Alteromonadales;f__Alteromonadaceae;g__Cellvibrio;s__ | 0.0006 | 0.0000 | 0.0025 | 0.0000 | 0.0000 |
| k__Bacteria;p__Proteobacteria;c__Gammaproteobacteria;o__Alteromonadales;f__[Chromatiaceae];g__Rheinheimera;s__ | 0.0010 | 0.0000 | 0.0000 | 0.0041 | 0.0000 |
| k__Bacteria;p__Proteobacteria;c__Gammaproteobacteria;o__Cardiobacteriales;f__Cardiobacteriaceae;g__Cardiobacterium;s__ | 0.0002 | 0.0000 | 0.0006 | 0.0000 | 0.0000 |
| k__Bacteria;p__Proteobacteria;c__Gammaproteobacteria;o__Cardiobacteriales;f__Cardiobacteriaceae;g__Suttonella;Other | 0.0001 | 0.0000 | 0.0000 | 0.0003 | 0.0000 |
| k__Bacteria;p__Proteobacteria;c__Gammaproteobacteria;o__Enterobacteriales;f__Enterobacteriaceae;Other;Other | 0.0003 | 0.0000 | 0.0006 | 0.0000 | 0.0005 |
| k__Bacteria;p__Proteobacteria;c__Gammaproteobacteria;o__Enterobacteriales;f__Enterobacteriaceae;g__;s__ | 0.1340 | 0.1969 | 0.1246 | 0.0703 | 0.1441 |
| k__Bacteria;p__Proteobacteria;c__Gammaproteobacteria;o__Enterobacteriales;f__Enterobacteriaceae;g__Citrobacter;s__ | 0.0001 | 0.0005 | 0.0000 | 0.0000 | 0.0000 |
| k__Bacteria;p__Proteobacteria;c__Gammaproteobacteria;o__Enterobacteriales;f__Enterobacteriaceae;g__Erwinia;Other | 0.0005 | 0.0010 | 0.0002 | 0.0009 | 0.0000 |
| k__Bacteria;p__Proteobacteria;c__Gammaproteobacteria;o__Enterobacteriales;f__Enterobacteriaceae;g__Erwinia;s__ | 0.0001 | 0.0000 | 0.0004 | 0.0000 | 0.0000 |
| k__Bacteria;p__Proteobacteria;c__Gammaproteobacteria;o__Enterobacteriales;f__Enterobacteriaceae;g__Klebsiella;s__ | 0.0009 | 0.0012 | 0.0010 | 0.0000 | 0.0012 |
| k__Bacteria;p__Proteobacteria;c__Gammaproteobacteria;o__Enterobacteriales;f__Enterobacteriaceae;g__Proteus;s__ | 0.0008 | 0.0000 | 0.0000 | 0.0033 | 0.0000 |
| k__Bacteria;p__Proteobacteria;c__Gammaproteobacteria;o__Enterobacteriales;f__Enterobacteriaceae;g__Providencia;Other | 0.0006 | 0.0000 | 0.0023 | 0.0000 | 0.0000 |
| k__Bacteria;p__Proteobacteria;c__Gammaproteobacteria;o__Enterobacteriales;f__Enterobacteriaceae;g__Trabulsiella;s__farmeri | 0.0001 | 0.0000 | 0.0000 | 0.0006 | 0.0000 |
| k__Bacteria;p__Proteobacteria;c__Gammaproteobacteria;o__Oceanospirillales;f__Halomonadaceae;g__Halomonas;s__ | 0.0023 | 0.0000 | 0.0000 | 0.0009 | 0.0082 |
| k__Bacteria;p__Proteobacteria;c__Gammaproteobacteria;o__Oceanospirillales;f__Oceanospirillaceae;Other;Other | 0.0001 | 0.0002 | 0.0000 | 0.0000 | 0.0000 |
| k__Bacteria;p__Proteobacteria;c__Gammaproteobacteria;o__Pasteurellales;f__Pasteurellaceae;g__;s__ | 0.0004 | 0.0002 | 0.0002 | 0.0005 | 0.0005 |
| k__Bacteria;p__Proteobacteria;c__Gammaproteobacteria;o__Pasteurellales;f__Pasteurellaceae;g__Actinobacillus;Other | 0.1609 | 0.2858 | 0.1242 | 0.1331 | 0.1005 |
| k__Bacteria;p__Proteobacteria;c__Gammaproteobacteria;o__Pasteurellales;f__Pasteurellaceae;g__Actinobacillus;s__ | 0.0282 | 0.0479 | 0.0151 | 0.0290 | 0.0207 |
| k__Bacteria;p__Proteobacteria;c__Gammaproteobacteria;o__Pasteurellales;f__Pasteurellaceae;g__Aggregatibacter;Other | 0.0032 | 0.0065 | 0.0005 | 0.0057 | 0.0000 |
| k__Bacteria;p__Proteobacteria;c__Gammaproteobacteria;o__Pasteurellales;f__Pasteurellaceae;g__Aggregatibacter;s__ | 0.0037 | 0.0000 | 0.0032 | 0.0065 | 0.0052 |
| k__Bacteria;p__Proteobacteria;c__Gammaproteobacteria;o__Pasteurellales;f__Pasteurellaceae;g__Aggregatibacter;s__segnis | 0.0005 | 0.0000 | 0.0004 | 0.0000 | 0.0015 |
| k__Bacteria;p__Proteobacteria;c__Gammaproteobacteria;o__Pasteurellales;f__Pasteurellaceae;g__Haemophilus;s__ | 0.0020 | 0.0064 | 0.0004 | 0.0008 | 0.0005 |
| k__Bacteria;p__Proteobacteria;c__Gammaproteobacteria;o__Pasteurellales;f__Pasteurellaceae;g__Haemophilus;s__parainfluenzae | 0.0029 | 0.0040 | 0.0008 | 0.0039 | 0.0029 |
| k__Bacteria;p__Proteobacteria;c__Gammaproteobacteria;o__Pasteurellales;f__Pasteurellaceae;g__Haemophilus;s__parasuis | 0.0001 | 0.0002 | 0.0000 | 0.0000 | 0.0000 |
| k__Bacteria;p__Proteobacteria;c__Gammaproteobacteria;o__Pseudomonadales;Other;Other;Other | 0.0001 | 0.0000 | 0.0000 | 0.0002 | 0.0000 |
| k__Bacteria;p__Proteobacteria;c__Gammaproteobacteria;o__Pseudomonadales;f__Moraxellaceae;g__;s__ | 0.0022 | 0.0002 | 0.0002 | 0.0055 | 0.0028 |
| k__Bacteria;p__Proteobacteria;c__Gammaproteobacteria;o__Pseudomonadales;f__Moraxellaceae;g__Acinetobacter;Other | 0.0001 | 0.0000 | 0.0000 | 0.0002 | 0.0000 |
| k__Bacteria;p__Proteobacteria;c__Gammaproteobacteria;o__Pseudomonadales;f__Moraxellaceae;g__Acinetobacter;s__ | 0.3759 | 0.2459 | 0.3771 | 0.8026 | 0.0781 |
| k__Bacteria;p__Proteobacteria;c__Gammaproteobacteria;o__Pseudomonadales;f__Moraxellaceae;g__Acinetobacter;s__johnsonii | 0.0170 | 0.0208 | 0.0232 | 0.0166 | 0.0073 |
| k__Bacteria;p__Proteobacteria;c__Gammaproteobacteria;o__Pseudomonadales;f__Moraxellaceae;g__Acinetobacter;s__lwoffii | 0.0118 | 0.0033 | 0.0117 | 0.0322 | 0.0000 |
| k__Bacteria;p__Proteobacteria;c__Gammaproteobacteria;o__Pseudomonadales;f__Moraxellaceae;g__Acinetobacter;s__rhizosphaerae | 0.0004 | 0.0000 | 0.0000 | 0.0017 | 0.0000 |
| k__Bacteria;p__Proteobacteria;c__Gammaproteobacteria;o__Pseudomonadales;f__Moraxellaceae;g__Acinetobacter;s__schindleri | 0.0006 | 0.0000 | 0.0005 | 0.0013 | 0.0004 |
| k__Bacteria;p__Proteobacteria;c__Gammaproteobacteria;o__Pseudomonadales;f__Moraxellaceae;g__Enhydrobacter;s__ | 0.0395 | 0.0084 | 0.0576 | 0.0756 | 0.0163 |
| k__Bacteria;p__Proteobacteria;c__Gammaproteobacteria;o__Pseudomonadales;f__Moraxellaceae;g__Moraxella;s__ | 0.7048 | 0.9076 | 0.6572 | 0.7099 | 0.5444 |
| k__Bacteria;p__Proteobacteria;c__Gammaproteobacteria;o__Pseudomonadales;f__Pseudomonadaceae;Other;Other | 0.0001 | 0.0002 | 0.0000 | 0.0000 | 0.0000 |
| k__Bacteria;p__Proteobacteria;c__Gammaproteobacteria;o__Pseudomonadales;f__Pseudomonadaceae;g__;s__ | 0.0023 | 0.0000 | 0.0087 | 0.0000 | 0.0003 |
| k__Bacteria;p__Proteobacteria;c__Gammaproteobacteria;o__Pseudomonadales;f__Pseudomonadaceae;g__Pseudomonas;Other | 0.0008 | 0.0000 | 0.0000 | 0.0033 | 0.0000 |
| k__Bacteria;p__Proteobacteria;c__Gammaproteobacteria;o__Pseudomonadales;f__Pseudomonadaceae;g__Pseudomonas;s__ | 0.0164 | 0.0264 | 0.0239 | 0.0148 | 0.0005 |
| k__Bacteria;p__Proteobacteria;c__Gammaproteobacteria;o__Pseudomonadales;f__Pseudomonadaceae;g__Pseudomonas;s__viridiflava | 0.0001 | 0.0000 | 0.0005 | 0.0000 | 0.0000 |
| k__Bacteria;p__Proteobacteria;c__Gammaproteobacteria;o__Xanthomonadales;f__Sinobacteraceae;g__;s__ | 0.0022 | 0.0038 | 0.0050 | 0.0000 | 0.0000 |
| k__Bacteria;p__Proteobacteria;c__Gammaproteobacteria;o__Xanthomonadales;f__Sinobacteraceae;g__Nevskia;s__ | 0.0008 | 0.0000 | 0.0033 | 0.0000 | 0.0000 |
| k__Bacteria;p__Proteobacteria;c__Gammaproteobacteria;o__Xanthomonadales;f__Sinobacteraceae;g__Steroidobacter;s__ | 0.0001 | 0.0000 | 0.0000 | 0.0003 | 0.0000 |
| k__Bacteria;p__Proteobacteria;c__Gammaproteobacteria;o__Xanthomonadales;f__Xanthomonadaceae;Other;Other | 0.0038 | 0.0023 | 0.0088 | 0.0042 | 0.0000 |
| k__Bacteria;p__Proteobacteria;c__Gammaproteobacteria;o__Xanthomonadales;f__Xanthomonadaceae;g__;s__ | 0.0056 | 0.0102 | 0.0104 | 0.0014 | 0.0005 |
| k__Bacteria;p__Proteobacteria;c__Gammaproteobacteria;o__Xanthomonadales;f__Xanthomonadaceae;g__Dokdonella;s__ | 0.0002 | 0.0000 | 0.0006 | 0.0000 | 0.0000 |
| k__Bacteria;p__Proteobacteria;c__Gammaproteobacteria;o__Xanthomonadales;f__Xanthomonadaceae;g__Luteimonas;s__ | 0.0042 | 0.0046 | 0.0002 | 0.0121 | 0.0000 |
| k__Bacteria;p__Proteobacteria;c__Gammaproteobacteria;o__Xanthomonadales;f__Xanthomonadaceae;g__Lysobacter;s__ | 0.0009 | 0.0000 | 0.0032 | 0.0003 | 0.0000 |
| k__Bacteria;p__Proteobacteria;c__Gammaproteobacteria;o__Xanthomonadales;f__Xanthomonadaceae;g__Pseudoxanthomonas;Other | 0.0006 | 0.0000 | 0.0000 | 0.0000 | 0.0023 |
| k__Bacteria;p__Proteobacteria;c__Gammaproteobacteria;o__Xanthomonadales;f__Xanthomonadaceae;g__Pseudoxanthomonas;s__mexicana | 0.0005 | 0.0000 | 0.0002 | 0.0000 | 0.0018 |
| k__Bacteria;p__Proteobacteria;c__Gammaproteobacteria;o__Xanthomonadales;f__Xanthomonadaceae;g__Stenotrophomonas;Other | 0.0044 | 0.0095 | 0.0071 | 0.0011 | 0.0000 |
| k__Bacteria;p__Proteobacteria;c__Gammaproteobacteria;o__Xanthomonadales;f__Xanthomonadaceae;g__Stenotrophomonas;s__ | 0.0003 | 0.0000 | 0.0011 | 0.0000 | 0.0000 |
| k__Bacteria;p__Proteobacteria;c__Gammaproteobacteria;o__Xanthomonadales;f__Xanthomonadaceae;g__Xanthomonas;Other | 0.0001 | 0.0000 | 0.0000 | 0.0006 | 0.0000 |
| k__Bacteria;p__Proteobacteria;c__Gammaproteobacteria;o__Xanthomonadales;f__Xanthomonadaceae;g__Xanthomonas;s__ | 0.0012 | 0.0046 | 0.0002 | 0.0000 | 0.0000 |
| k__Bacteria;p__Spirochaetes;c__Spirochaetes;o__Sphaerochaetales;f__Sphaerochaetaceae;g__Sphaerochaeta;s__ | 0.0006 | 0.0000 | 0.0007 | 0.0005 | 0.0014 |
| k__Bacteria;p__Spirochaetes;c__Spirochaetes;o__Spirochaetales;f__Spirochaetaceae;g__Treponema;s__ | 0.8452 | 0.7833 | 0.8479 | 0.9185 | 0.8312 |
| k__Bacteria;p__Spirochaetes;c__Spirochaetes;o__Spirochaetales;f__Spirochaetaceae;g__Treponema;s__socranskii | 0.0001 | 0.0000 | 0.0002 | 0.0000 | 0.0000 |
| k__Bacteria;p__Synergistetes;c__Synergistia;o__Synergistales;f__Dethiosulfovibrionaceae;g__;s__ | 0.0007 | 0.0000 | 0.0000 | 0.0002 | 0.0024 |
| k__Bacteria;p__Synergistetes;c__Synergistia;o__Synergistales;f__Dethiosulfovibrionaceae;g__Pyramidobacter;s__piscolens | 0.0165 | 0.0031 | 0.0178 | 0.0285 | 0.0165 |
| k__Bacteria;p__Synergistetes;c__Synergistia;o__Synergistales;f__Synergistaceae;Other;Other | 0.0016 | 0.0008 | 0.0039 | 0.0000 | 0.0015 |
| k__Bacteria;p__Synergistetes;c__Synergistia;o__Synergistales;f__Synergistaceae;g__;s__ | 0.0002 | 0.0007 | 0.0000 | 0.0000 | 0.0000 |
| k__Bacteria;p__TM7;c__TM7-1;o__;f__;g__;s__ | 0.0001 | 0.0000 | 0.0000 | 0.0002 | 0.0000 |
| k__Bacteria;p__TM7;c__TM7-3;o__;f__;g__;s__ | 0.0003 | 0.0000 | 0.0009 | 0.0002 | 0.0000 |
| k__Bacteria;p__TM7;c__TM7-3;o__CW040;f__;g__;s__ | 0.0010 | 0.0030 | 0.0000 | 0.0012 | 0.0000 |
| k__Bacteria;p__TM7;c__TM7-3;o__CW040;f__F16;g__;s__ | 0.0034 | 0.0000 | 0.0088 | 0.0005 | 0.0042 |
| k__Bacteria;p__Tenericutes;c__Mollicutes;o__Anaeroplasmatales;f__Anaeroplasmataceae;g__;s__ | 0.0477 | 0.0641 | 0.0249 | 0.0538 | 0.0478 |
| k__Bacteria;p__Tenericutes;c__Mollicutes;o__Anaeroplasmatales;f__Anaeroplasmataceae;g__Anaeroplasma;s__ | 0.0987 | 0.1070 | 0.0621 | 0.1152 | 0.1106 |
| k__Bacteria;p__Tenericutes;c__Mollicutes;o__Mycoplasmatales;f__Mycoplasmataceae;g__Mycoplasma;s__ | 0.0061 | 0.0099 | 0.0137 | 0.0009 | 0.0000 |
| k__Bacteria;p__Tenericutes;c__Mollicutes;o__Mycoplasmatales;f__Mycoplasmataceae;g__Mycoplasma;s__hyorhinis | 0.0347 | 0.0709 | 0.0384 | 0.0103 | 0.0193 |
| k__Bacteria;p__Tenericutes;c__Mollicutes;o__RF39;f__;g__;s__ | 0.3441 | 0.4407 | 0.1980 | 0.3506 | 0.3871 |
| k__Bacteria;p__Tenericutes;c__Mollicutes;o__RsaHF231;f__;g__;s__ | 0.0003 | 0.0000 | 0.0000 | 0.0000 | 0.0012 |
| k__Bacteria;p__Tenericutes;c__RF3;o__ML615J-28;f__;g__;s__ | 0.0083 | 0.0012 | 0.0080 | 0.0149 | 0.0093 |
| k__Bacteria;p__Verrucomicrobia;c__Opitutae;o__[Cerasicoccales];f__[Cerasicoccaceae];g__;s__ | 0.0161 | 0.0005 | 0.0120 | 0.0260 | 0.0259 |
| k__Bacteria;p__Verrucomicrobia;c__Verruco-5;o__WCHB1-41;f__RFP12;g__;s__ | 0.2038 | 0.1011 | 0.1724 | 0.2386 | 0.3031 |
| k__Bacteria;p__Verrucomicrobia;c__Verruco-5;o__WCHB1-41;f__WCHB1-25;g__;s__ | 0.0599 | 0.0491 | 0.0649 | 0.0435 | 0.0821 |
| k__Bacteria;p__Verrucomicrobia;c__Verrucomicrobiae;o__Verrucomicrobiales;f__Verrucomicrobiaceae;g__;s__ | 0.0004 | 0.0000 | 0.0000 | 0.0000 | 0.0016 |
| k__Bacteria;p__Verrucomicrobia;c__Verrucomicrobiae;o__Verrucomicrobiales;f__Verrucomicrobiaceae;g__Akkermansia;s__ | 0.0405 | 0.0492 | 0.0399 | 0.0407 | 0.0323 |
| k__Bacteria;p__Verrucomicrobia;c__[Pedosphaerae];o__[Pedosphaerales];f__;g__;s__ | 0.0002 | 0.0000 | 0.0000 | 0.0000 | 0.0009 |
| k__Bacteria;p__WPS-2;c__;o__;f__;g__;s__ | 0.0284 | 0.0353 | 0.0166 | 0.0302 | 0.0315 |
| k__Bacteria;p__[Thermi];c__Deinococci;o__Deinococcales;f__Deinococcaceae;g__Deinococcus;s__ | 0.0036 | 0.0000 | 0.0053 | 0.0091 | 0.0000 |
| k__Bacteria;p__[Thermi];c__Deinococci;o__Thermales;f__Thermaceae;g__Thermus;s__ | 0.0004 | 0.0000 | 0.0017 | 0.0000 | 0.0000 |
