# Supplementary material for: Initial Characterization of the Pig Skin Bacteriome and Its Effect on In Vitro Models of Wound Healing
Source: PLoS One. 2016 Nov 8;11(11):e0166176. doi: 10.1371/journal.pone.0166176 (PMC5100914; doi:10.1371/journal.pone.0166176)
Supplement: S2 Table — Species-level analysis of differences comparing all swab sites. Only species with significant differences (2-way ANOVA with Tukey’s Post-testing, n = 4/AL, p<0.05) are shown. (DOCX) [file pone.0166176.s004.docx]

|  | AL1 vs. AL2 | | | AL1 vs. AL3 | | | AL1 vs. AL4 | | | AL2 vs. AL3 | | | AL2 vs. AL4 | | | AL3 vs. AL4 | | |
| --- | --- | --- | --- | --- | --- | --- | --- | --- | --- | --- | --- | --- | --- | --- | --- | --- | --- | --- |
| Bacteria | % AL1 | % AL2 | P-value | % AL1 | % AL3 | P-value | % AL1 | % AL4 | P-value | %AL2 | %AL3 | P-value | %AL2 | %AL4 | P-value | %AL3 | %AL4 | P-value |
| Brachybacterium;s__ | 0.31 | 1.68 | 0.014 | 0.31 | 1.88 | 0.003 | 0.31 | 0.12 | ns | 1.68 | 1.88 | ns | 1.68 | 0.12 | 0.004 | 1.88 | 0.12 | 0.001 |
| *Kocuria rhizophila* | 2.36 | 12.72 | < 0.0001 | 2.36 | 10.87 | < 0.0001 | 2.36 | 2.01 | ns | 12.72 | 10.87 | 0.0003 | 12.72 | 2.01 | < 0.0001 | 10.87 | 2.01 | < 0.0001 |
| Micrococcus;s__ | 0.80 | 4.14 | < 0.0001 | 0.80 | 5.11 | < 0.0001 | 0.80 | 0.20 | ns | 4.14 | 5.11 | Ns | 4.14 | 0.20 | < 0.0001 | 5.11 | 0.20 | < 0.0001 |
| o__Bacteroidales;f__;g__;s__ | 2.97 | 1.88 | ns | 2.97 | 3.74 | ns | 2.97 | 4.51 | 0.004 | 1.88 | 3.74 | 0.0003 | 1.88 | 4.51 | < 0.0001 | 3.74 | 4.51 | ns |
| Prevotella;s__ | 7.73 | 5.70 | < 0.0001 | 7.73 | 6.40 | 0.019 | 7.73 | 8.78 | ns | 5.70 | 6.40 | ns | 5.70 | 8.78 | < 0.0001 | 6.40 | 8.78 | < 0.0001 |
| Prevotella copri | 2.65 | 1.26 | 0.013 | 2.65 | 2.53 | ns | 2.65 | 2.98 | ns | 1.26 | 2.53 | 0.028 | 1.26 | 2.98 | 0.001 | 2.53 | 2.98 | ns |
| f__[Paraprevotellaceae];g__;s__ | 0.98 | 0.56 | ns | 0.98 | 1.97 | ns | 0.98 | 1.54 | ns | 0.56 | 1.97 | 0.011 | 0.56 | 1.54 | ns | 1.97 | 1.54 | ns |
| Lactobacillus;s__ | 0.01 | 0.00 | 0.003 | 0.01 | 0.00 | < 0.0001 | 0.01 | 0.00 | < 0.0001 | 0.00 | 0.00 | ns | 0.00 | 0.00 | ns | 0.00 | 0.00 | ns |
| Streptococcus;s__ | 3.41 | 2.53 | ns | 3.41 | 1.11 | < 0.0001 | 3.41 | 2.59 | ns | 2.53 | 1.11 | 0.010 | 2.53 | 2.59 | ns | 1.11 | 2.59 | 0.006 |
| ;f__Clostridiaceae;g__;s__ | 6.33 | 3.50 | < 0.0001 | 6.33 | 0.63 | < 0.0001 | 6.33 | 1.06 | < 0.0001 | 3.50 | 0.63 | < 0.0001 | 3.50 | 1.06 | < 0.0001 | 0.63 | 1.06 | ns |
| f__Lachnospiraceae;g__;s__ | 5.82 | 6.61 | ns | 5.82 | 6.09 | ns | 5.82 | 10.90 | < 0.0001 | 6.61 | 6.09 | ns | 6.61 | 10.90 | < 0.0001 | 6.09 | 10.90 | < 0.0001 |
| g__Lachnospira;s__ | 1.91 | 1.38 | ns | 1.91 | 1.95 | ns | 1.91 | 3.35 | 0.009 | 1.38 | 1.95 | ns | 1.38 | 3.35 | < 0.0001 | 1.95 | 3.35 | 0.012 |
| f__Ruminococcaceae;g__;s__ | 10.53 | 11.46 | ns | 10.53 | 10.12 | ns | 10.53 | 12.96 | < 0.0001 | 11.46 | 10.12 | 0.017 | 11.46 | 12.96 | 0.006 | 10.12 | 12.96 | < 0.0001 |
| Faecalibacterium prausnitzii | 1.98 | 1.98 | ns | 1.98 | 3.05 | ns | 1.98 | 4.08 | < 0.0001 | 1.98 | 3.05 | ns | 1.98 | 4.08 | < 0.0001 | 3.05 | 4.08 | ns |
